# The Cardiovascular Benefits and Infections Risk of SGLT2i versus Metformin in Type 2 Diabetes: A Systemic Review and Meta-Analysis

**DOI:** 10.3390/metabo12100979

**Published:** 2022-10-17

**Authors:** Chunmei Xu, Liping He, Jing Zhang, Lusi Xu, Jianjun Dong, Lin Liao

**Affiliations:** 1 Department of Endocrinology, Shandong Provincial Hospital Affiliated to Shandong First Medical University, Jinan 250021, China; 2 Shandong Key Laboratory of Endocrinology and Lipid Metabolism, Shandong Provincial Hospital, Jinan 250021, China; 3 Department of Endocrinology, Shandong Provincial Hospital, Shandong University, Jinan 250021, China; 4 Department of Endocrinology and Metabology, The First Affiliated Hospital of Shandong First Medical University & Shandong Provincial Qianfoshan Hospital, Shandong Key Laboratory of Rheumatic Disease and Translational medicine, Shandong Institute of Nephrology, Jinan 250014, China; 5 Department of Endocrinology and Metabology, Shandong Provincial Qianfoshan Hospital, Shandong University, Jinan 250014, China; 6 Division of Endocrinology, Department of Internal Medicine, Qilu Hospital of Shandong University, Jinan 250012, China

**Keywords:** sodium-glucose cotransporter 2 inhibitors, genitourinary tract infections, cardiovascular benefits, metformin, randomized controlled trials, meta-analysis

## Abstract

Sodium-glucose cotransporter 2 inhibitors (SGLT2i) and metformin are both widely accepted anti-hyperglycemic agents. However, there is still no systematic review evaluating the cardiovascular benefits and risk of infections of SGLT2i versus metformin. To make that clear, we designed this study. Public databases, including the Cochrane library database, PubMed, and Embase were searched for randomized clinical trials (RCTs) fitting the inclusion criteria. Two reviewers extracted the data and appraised the study quality independently. Thirteen RCTs enrolling 4189 patients were eligible for this analysis. Our results showed that compared with metformin, SGLT2i increased the risk of genitourinary tract infections (*p* < 0.00001). Further subgroup analysis suggested that the occurrence of urinary tract infections (UTI) was not statistically significant (*p* = 0.18), but the incidence of reproductive tract infections (RTI) was significantly increased in patients in the SGLT2i group compared with that in the metformin group (*p* < 0.00001). In addition, SGLT2i markedly decreased the levels of cardiovascular risk factor, including body weight, blood pressure, and triglyceride level, and significantly increased the HDL-cholesterol level (*p* < 0.00001) in patients versus that of metformin. For type 2 diabetes patients with obesity, SGLT2i was associated with more significant reductions in weight and blood pressure compared to metformin without an increased risk of genitourinary infections, and the reduction in fasting plasma glucose was superior in the SGLT2i group; the decrease in HbA1c was similar in both groups. Additionally, no significant publication bias was seen. Based on these findings, SGLT2i provided the similar antihyperglycemic effects, additional cardiovascular benefits, and a potential RTI risk compared with that of metformin. Our results indicate that SGLT2i is a good choice for those patients with metformin intolerance or resistance.

## 1. Introduction

Diabetes mellitus which acts as one of the most common chronic diseases has caused a major public health crisis worldwide due to its high prevalence [1,2]. According to a recent cross-sectional study, type 2 diabetes mellitus (T2DM) accounts for the majority of patients in China, and the increasing proportion of young patients leads to a high morbidity, distinct from other chronic diseases and mortality [3]. Studies have found that the hazard ratio for death due to cardiovascular disease in diabetic patients is increasing year by year, and now cardiovascular disease has become the main cause of death in T2DM patients [4]. Reducing the incidence of cardiovascular disease (CVD) is so vital for T2DM patients that the American Diabetes Association recommends a glucose-lowering drug which could provide additional cardiovascular safety as a prerequisite [5]. At present, many guidelines recommend sodium-glucose cotransporter 2 inhibitors (SGLT2i) as a priority for diabetics with atherosclerotic cardiovascular disease (ASCVD).

SGT2i is an effective and widely used oral antidiabetic drug which can significantly decrease hyperglycemia by increasing urinary glucose excretion, independent of the pancreatic β cell function [6,7,8,9]. SGLT2i has been widely used due to its unique advantage in weight loss, renal protection, and cardiovascular benefits [10,11]. SGLT2i may be regarded as the top option of pharmacotherapy that raises new healthcare decision-making both for clinicians and policy makers. Nonetheless, large amounts of clinical trials worldwide are concerned regarding the safety of SGLT2i in patients with T2DM, as they are potentially causing urinary tract infections (UTI) and reproductive tract infections (RTI) [12,13,14]. In addition, sporadic skin and respiratory infections may also induce the aggravation of T2DM, which affects the quality of life of the patients. So far, there are many articles reporting the outcomes of cardiovascular and urinary systems of SGLT2i, however, most of them analyzed the benefits of SGLT2i as an add-on treatment for metformin compared with placebo [12,15,16,17,18]. Rare reports were available comparing the cardiovascular outcomes and safety of SGLT2i monotherapy with the first-line antihyperglycemic drug, metformin. Therefore, we sought to provide a comprehensive new estimate of the cardiovascular benefits, risk of infection, and glycemic efficacy of SGLT2i in T2DM patients when compared with metformin monotherapy. In view of the limited data on cardiovascular outcomes, we performed an analysis of cardiovascular risk factors as a substitute for endpoint assessment, including body weight, total cholesterol, HDL-cholesterol, LDL-cholesterol, triglycerides, diastolic blood pressure, and systolic blood pressure [19]. It is expected to provide evidence for doctors to choose whether SGLT2i or metformin will be preferred and it will be helpful for healthcare decision-making in the future.

## 2. Materials and Methods

### 2.1. Data Sources and Searches

In this meta-analysis of intervention studies, we performed a systematic search of the scientific literatures according to the PRISMA statement (PROSPERO registration number: CRD42022359007). A systematic search was conducted in the Cochrane Library database, Web of Science, Embase, PubMed, China National Knowledge Infrastructure (CNKI), and Wanfang Database for scientific literatures by two independent investigators using the same search strategy, collecting all randomized clinical trials (RCTs) on humans from inception to March 1st, 2022, with no other restrictions. The relevant text words and medical subject headings comprised terms relating to “Sodium-Glucose Transporter 2” and all the idiographic drug names, metformin, and diabetes mellitus (search strategy is provided in the Supplementary File S1). Furthermore, we also searched completed studies with the drugs specified above in the www.clinicaltrials.gov register to identify possible inclusion trials.

### 2.2. Study Selection

Two investigators independently assessed the articles by title and abstract, and studies that satisfied the inclusion criteria were retrieved for a full-text assessment, with disagreements resolved by mutual discussion.

Studies were included if the following inclusion criteria were met: (1) RCTs; (2) assessing the effects of any SGLT2i compared with metformin agent in humans with diabetes mellitus; (3) reported at least one type of infections (genitourinary tract infections, UTIs, RTIs, or other infection events); (4) reported at least one cardiometabolic or safety outcome (body weight, total cholesterol, HDL-cholesterol, LDL-cholesterol, triglycerides, diastolic blood pressure, and systolic blood pressure level); and (5) reported blood glucose level, including glycosylated hemoglobin (HbA1c) or fasting plasma glucose (FPG). The maximum non-overlapping data were extracted if different reports on the same trial provided data on different outcomes. Likewise, there were multiple reports of a single study and the report with longest follow-up was included.

Studies were excluded if they met any of the following exclusion criteria: (1) duplicate reports; (2) non-clinical studies or randomized controlled trials; and (3) did not report the interested outcomes (genitourinary tract infection events, effect on cardiovascular system and lowering glucose, and safety outcomes).

### 2.3. Data Extraction and Quality Assessment

Prespecified data from each trial were performed independently by two investigators using a standardized data collection form and disagreements were resolved by mutual discussion. The following items were included: first author, year of journal article publication, clinical trial registration number, number of participants, intervention and dose, study duration, gender distribution, baseline HbA1c and body weight, and other outcome measures listed below. A greater quantity of data provided by the www.clinicaltrials.gov register could be used to supplement missing data that were not reported sufficiently or not published at all from the original text. The Cochrane risk-of-bias tool for the randomized trials (Review Manager, version 5.2) was used to assess the methodological quality of the included trial. The specific assessment items of Cochrane definitions included random sequence generation (selection bias), allocation concealment (selection bias), blinding of participants and personnel (performance bias), blinding of outcome assessors (detection bias), incomplete outcome data (attrition bias), and selective outcome reporting (reporting bias). Two of the investigators performed the quality assessment, and disagreements were resolved by discussions. Funnel plots and the Egger statistical test were generated for exploring the risk of publication bias across the studies. 

### 2.4. Outcome Indicators

We grouped outcomes into four broad sets: (1) incidence of infection events; (2) effects on cardiovascular risk factors; (3) efficacy on glycemic control; and (4) incidence of hypoglycemic adverse events. The primary outcome of interest was the incidence of infections, including urinary or reproductive tract infection events and upper respiratory tract infections. Cardiovascular risk factors enrolled in this analysis included body weight, total cholesterol, HDL-cholesterol, LDL-cholesterol, triglycerides, diastolic blood pressure, and systolic blood pressure, and these indicators were analyzed by the change from baseline. The value changes in FPG and HbA1c were used to assess the effect of SGLT2i in lowering glucose.

### 2.5. Data Synthesis and Analysis

Statistical analyses were performed with RevMan 5.4 and Stata software, version 16.0 (StataCorp, College Station, TX, USA). For dichotomous variables, relative risk (RR) with a 95% confidence interval (CI) was used to present the pooled results, and weighed mean differences (WMD) with a 95% CI were calculated for the continuous variables. The analyses were calculated with fixed-effect models when there was no significant extent of heterogeneities. Conversely, random-effects models were used. When the heterogeneities were inevitable, a prior subgroup analysis was conducted, including types of SGLT2i and different doses of the drug, treatment periods (12 weeks, 24–26 weeks, and ≥52 weeks), and obesity (mean BMI > 28 Kg/m^2^) or not. The gender subgroup analysis of genitourinary tract infection events was performed for a different physiological structure of the genitourinary tract between male and female. Statistical heterogeneity was assessed with the *p*-value of v2-based Q test (cutoff value *p* = 0.10) and the I^2^ statistic (a low likelihood: 0–25%; moderate likelihood: 26–75%; and a high likelihood: 76–100%). Sensitivity analysis was performed by omitting each study sequentially to evaluate the robustness of the results of the pooled estimates. Two-tailed *p* values < 0.05 indicated statistically significant results.

## 3. Results

### 3.1. Search Results

**Figure 1:**
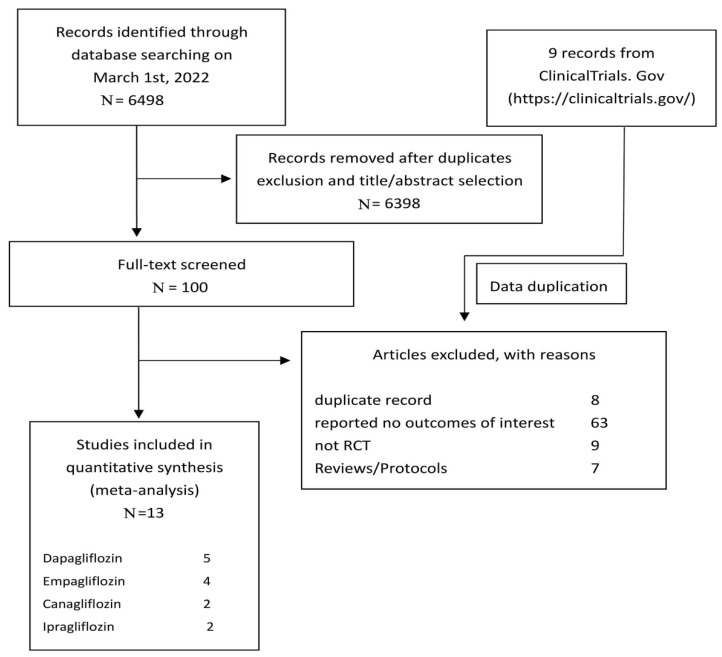
Flowchart for identification and selection of included trials.

Our research yielded 6498 potentially eligible studies, and 6398 articles were excluded by scanning the titles and abstracts. Eighty-seven studies were excluded because of improper article type or an inappropriate intervention or outcome measure, thus ultimately a total of 13 fulfilling studies [20,21,22,23,24,25,26,27,28,29,30,31,32] were identified for the meta-analysis (Figure 1). 

### 3.2. Study Characteristics 

Our search identified four types of SGLT2i with available data, including four studies of empagliflozin [20,21,22,23], five of dapagliflozin [24,25,26,27,28], two of canagliflozin [29,30], and two of ipragliflozin [31,32]. The baseline characteristics of the enrolled patients and the drug therapy information are illustrated in Table 1. A total of 4189 patients with T2DM were included in our study, among which 2699 patients were included in the SGLT2i group and 1490 patients in the metformin group. Among the 4189 patients, 9.24% (387/4189) cases had genitourinary tract infections, 5.67% (234/4127) cases had a UTI, 3.65% (140/3831) cases had an RTI, and 3.60% (92/2559) cases had upper respiratory tract infections. Among those patients, 57.13% (2393/4189) had their BMI >28 Kg/m^2^ [22,25,29,31]. In the study conducted by Araki, E. et al. in 2015 [20], in which the patients underwent a background treatment with sulfonylurea, and in the study conducted by Koshizaka, M. et al. [32] in 2019, where patients underwent a background treatment with DPP-4i, both applied SGLT2i and metformin for monotherapy. In the trial conducted by Pian Liu et al. in 2021 [27] and Jingqian Xie et al. in 2020 [30], all participants received routine congestive heart failure treatment.

### 3.3. Methodological Quality

**Figure 2:**
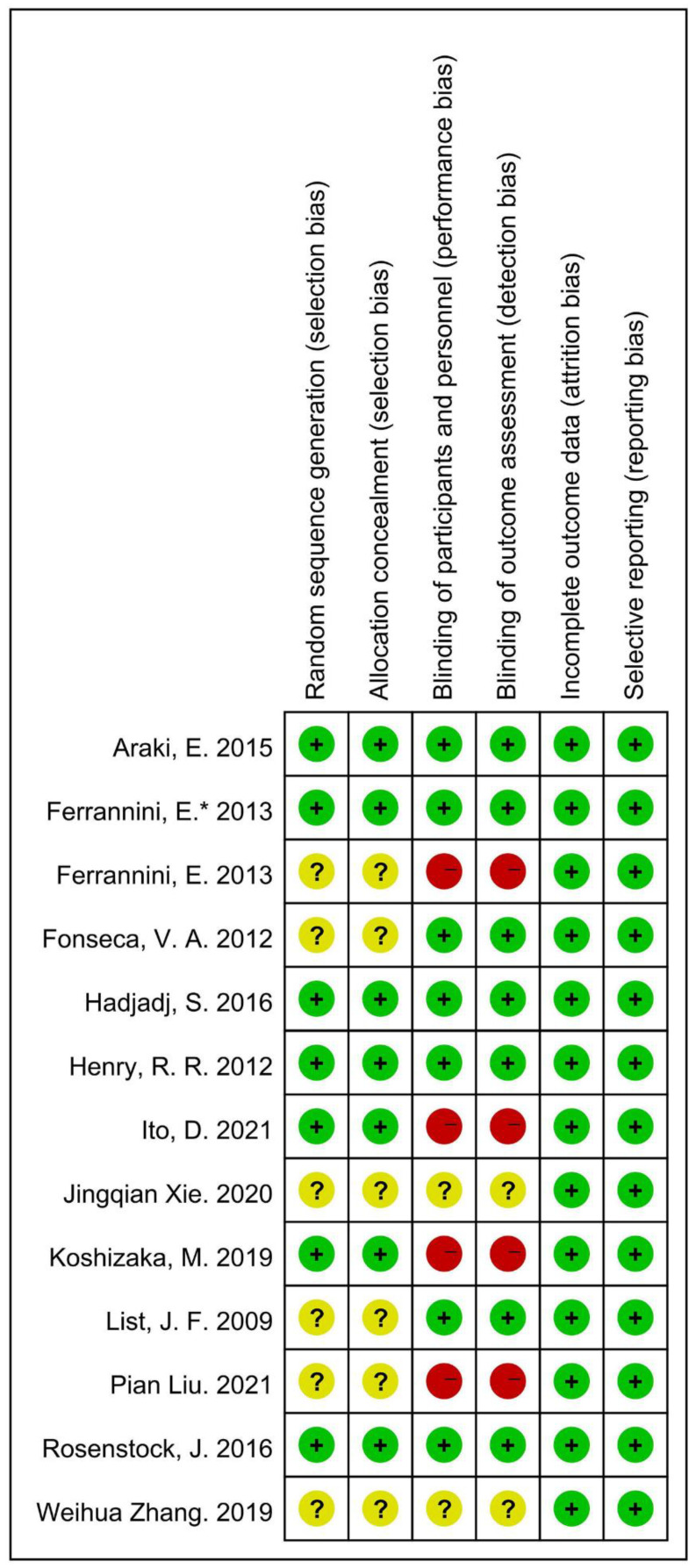
Figure for methodological quality of included trials [20,21,22,23,24,25,26,27,28,29,30,31,32]. Green symbol: low risk of bias; Yellow symbol: unclear risk of bias; Red symbol: high risk of bias.

For random sequence generation, the specific method was clearly presented in seven studies [20,21,22,24,26,29,32], but the remaining six referred to “random” without a detailed method [23,25,27,28,30,31]. Six studies mentioned the specific allocation concealment method [20,21,22,24,26,32]. The blinding of the participants and personnel was concretely presented in seven studies [20,21,22,24,25,29,31], and four studies were open-label [23,26,27,32]. Blinding was not mentioned in the 2019 Weihua Zhang et al. trial [28] nor the 2020 Jingqian Xie et al. trial [30]. Significantly, the control group (metformin) was open-label in Araki, E. 2015 [20] and Ferrannini, E.* 2013 [21], and double-blinding was presented in the experimental groups. Six studies had an incomplete outcome data bias [22,23,24,25,29,31]; the remaining seven studies did not describe this bias [20,21,26,27,28,30,32]. None of included studies mentioned the reporting bias. The results of the methodological quality are graphically displayed in Figure 2.

### 3.4. Outcomes

#### 3.4.1. Infection Incidence Risk

Genitourinary Tract Infections

**Figure 3:**
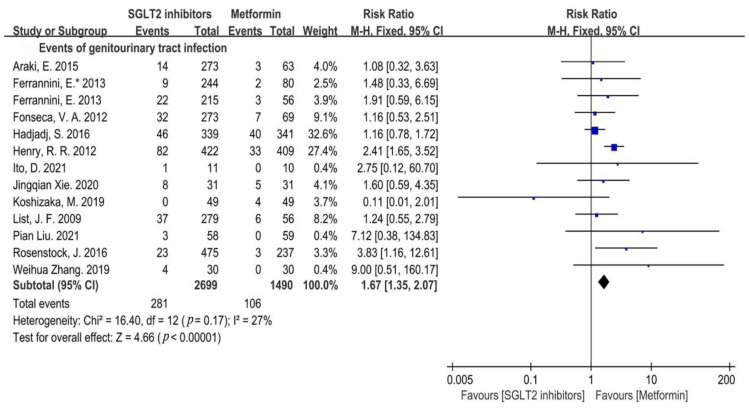
Risk of genitourinary tract infections of SGLT2i compared with that of metformin [20,21,22,23,24,25,26,27,28,29,30,31,32].

Genitourinary tract infections, one of the most common adverse effects of SGLT2i, were reported in all of the included RCTs. Compared with metformin, SGLT2i increased the risk of genitourinary tract infections (RR = 1.67, 95%CI = 1.35 to 2.07, *p* < 0.00001, I^2^ = 27%, Q test: *p* = 0.17) (Figure 3). 

**Figure 4:**
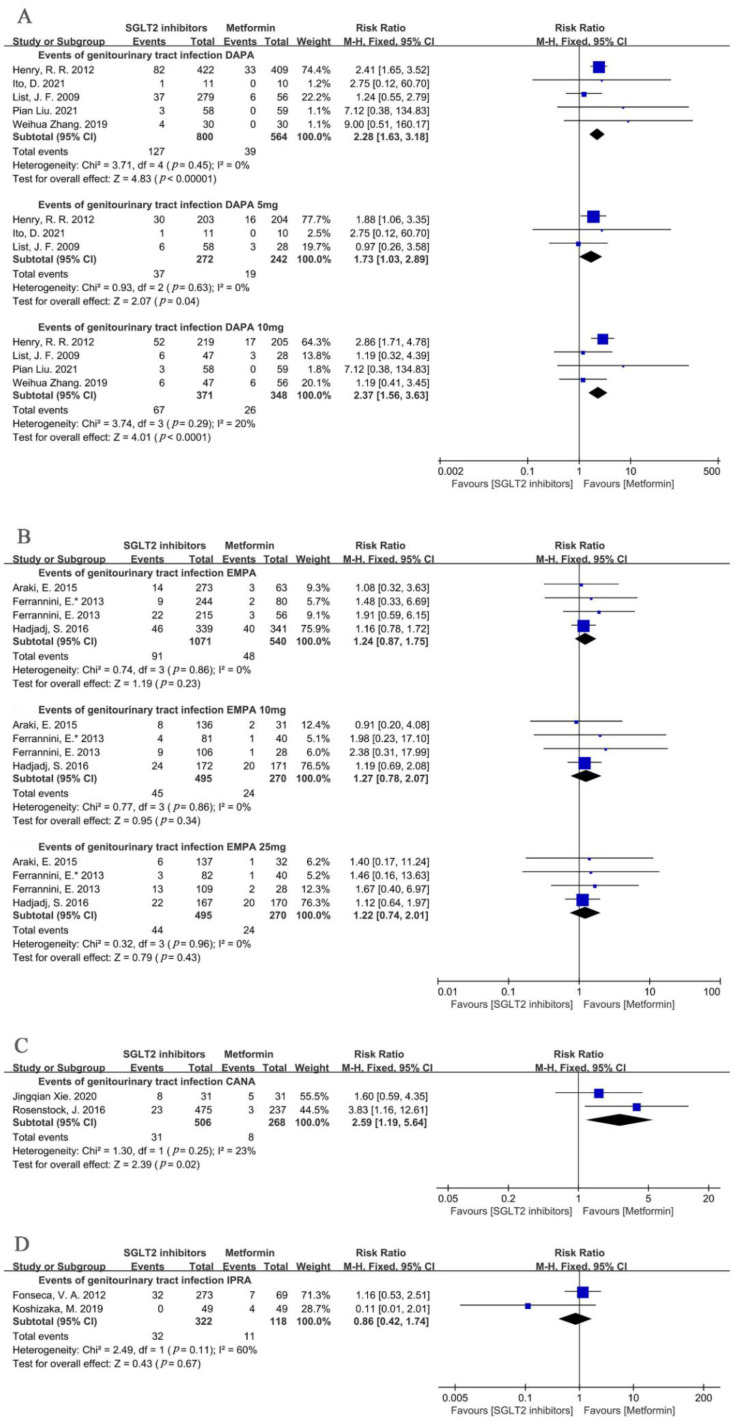
(**A**) Risk of genitourinary tract infections of DAPA and its different doses compared with metformin [24,25,26,27,28]. (**B**) Risk of genitourinary tract infections of EMPA and its different doses compared with metformin [20,21,22,23]. (**C**) Risk of genitourinary tract infections of CANA compared with metformin [29,30]. (**D**) Risk of genitourinary tract infections of IPRA compared with metformin [31,32]. DAPA = dapagliflozin. EMPA = empagliflozin. CANA = canagliflozin. IPRA = ipragliflozin.

A further subgroup analysis of the different types of SGLT2i suggested that dapagliflozin was associated with a significant increase in the incidence of genitourinary tract infections in comparison with that of metformin (dapagliflozin: RR = 2.28, 95%CI = 1.63 to 3.18, *p* < 0.00001, I^2^ = 0%, N = 5) (Figure 4A). Additionally, the different doses of dapagliflozin all showed a significant increase in the incidence of genitourinary tract infections in the SGLT2i group compared to those in the metformin group (dapagliflozin 5 mg: RR = 1.73, 95% = 1.03 to 2.89, *p* = 0.04, I^2^ = 0%, N = 3; dapagliflozin 10 mg: RR = 2.37, 95%CI = 1.56 to 3.63, *p* < 0.0001, I^2^ = 20%, N = 4) (Figure 4A).

There was not any significant difference between empagliflozin and metformin (empagliflozin: RR = 1.24, 95%CI = 0.87 to 1.75, *p* = 0.23, I^2^ = 0%, N = 4) (Figure 4B). The following subgroup analyses of the different doses of empagliflozin versus metformin showed consistent results (empagliflozin 10 mg: RR = 1.27, 95%CI = 0.78 to 2.07, *p* = 0.34, I^2^ = 0%, N = 4; empagliflozin 25 mg: RR = 1.22, 95%CI = 0.74 to 2.01, *p* = 0.43, I^2^ = 0%, N = 4) (Figure 4B).

In addition, the subgroup analysis of canagliflozin showed a significantly increased risk of genitourinary tract infections (RR = 2.59, 95%CI = 1.19 to 5.64, *p* = 0.02, I^2^ = 23%, N = 2) (Figure 4C). Ipragliflozin had a similar risk increase in genitourinary tract infections, despite the moderate heterogeneity (RR = 0.86, 95%CI = 0.42 to 1.74, *p* = 0.67, I^2^ = 60%, N = 2) (Figure 4D).

Urinary Tract Infections (UTI)

**Figure 5:**
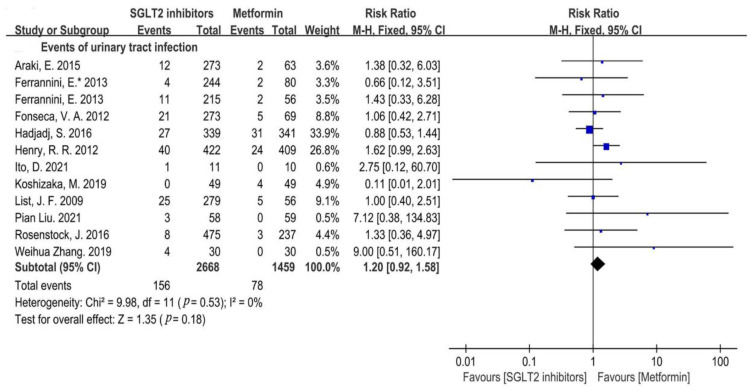
Risk of urinary tract infections in patients with SGLT2i compared with that of metformin [20,21,22,23,24,25,26,27,28,29,31,32].

All trials included in our meta-analysis reported the specific type of genitourinary tract infections, including UTIs and RTIs, except for one research conducted by Jingqian Xie et al. in a 2020 trial, which only reported genitourinary tract infection events [30]. Our analysis showed that the overall occurrence of UTIs was not statistically significant between the SGLT2i and metformin group (RR = 1.20, 95%CI = 0.92 to 1.58, *p* = 0.18, I^2^ = 0%, N = 12) (Figure 5). 

**Figure 6:**
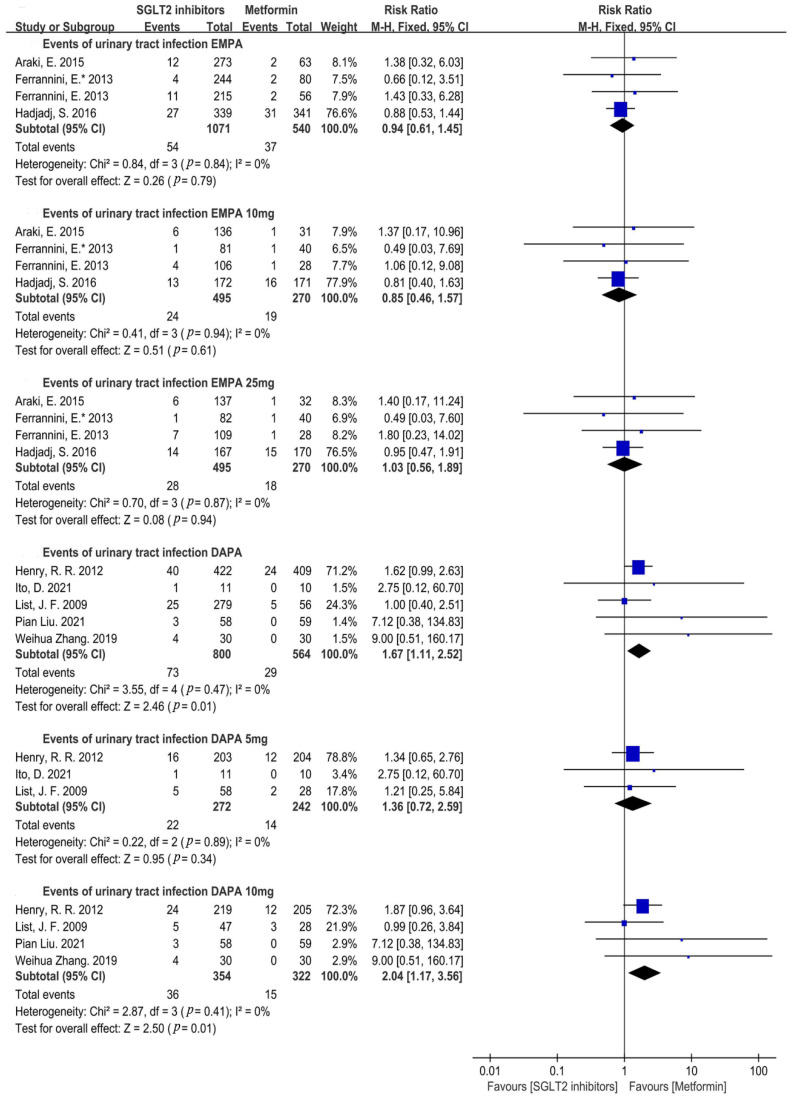
Risk of urinary tract infections with different types and doses of SGLT2i compared with that of metformin [20,21,22,23,24,25,26,27,28]. DAPA = dapagliflozin, EMPA = empagliflozin.

Drug types and doses were further investigated and found that there was no significant difference in the empagliflozin group compared to the metformin group, and both a low dose (10 mg) and high dose (25 mg) of empagliflozin showed a similar result (empagliflozin: RR = 0.94, 95%CI = 0.61 to 1.45, *p* = 0.79, I^2^ = 0%, N = 4; empagliflozin 10 mg: RR = 0.85, 95%CI = 0.46 to 1.57, *p* = 0.61, I^2^ = 0%, N = 4; empagliflozin 25 mg: RR = 1.03, 95%CI = 0.56 to 1.89, *p* = 0.94, I^2^ = 0%, N = 4) (Figure 6). Dapagliflozin resulted in a higher risk of a UTI compared to that of metformin (RR = 1.67, 95%CI = 1.11 to 2.52, *p* = 0.01, I^2^ = 0%, N = 5) (Figure 6). As for the influence of the dose, 10 mg of dapagliflozin significantly increased the risk of a UTI, but 5 mg of dapagliflozin had a similar risk of a UTI compared with that of metformin (dapagliflozin 5 mg: RR = 1.36, 95%CI = 0.72 to 2.59, *p* = 0.34, I^2^ = 0%, N = 3; dapagliflozin 10 mg: RR = 2.04, 95%CI = 1.17 to 3.56, *p* = 0.01, I^2^ = 0%, N = 4) (Figure 6). Only two RCTs reported canagliflozin and two RCTs reported ipragliflozin, so sub-group analyses were not performed.

**Figure 7:**
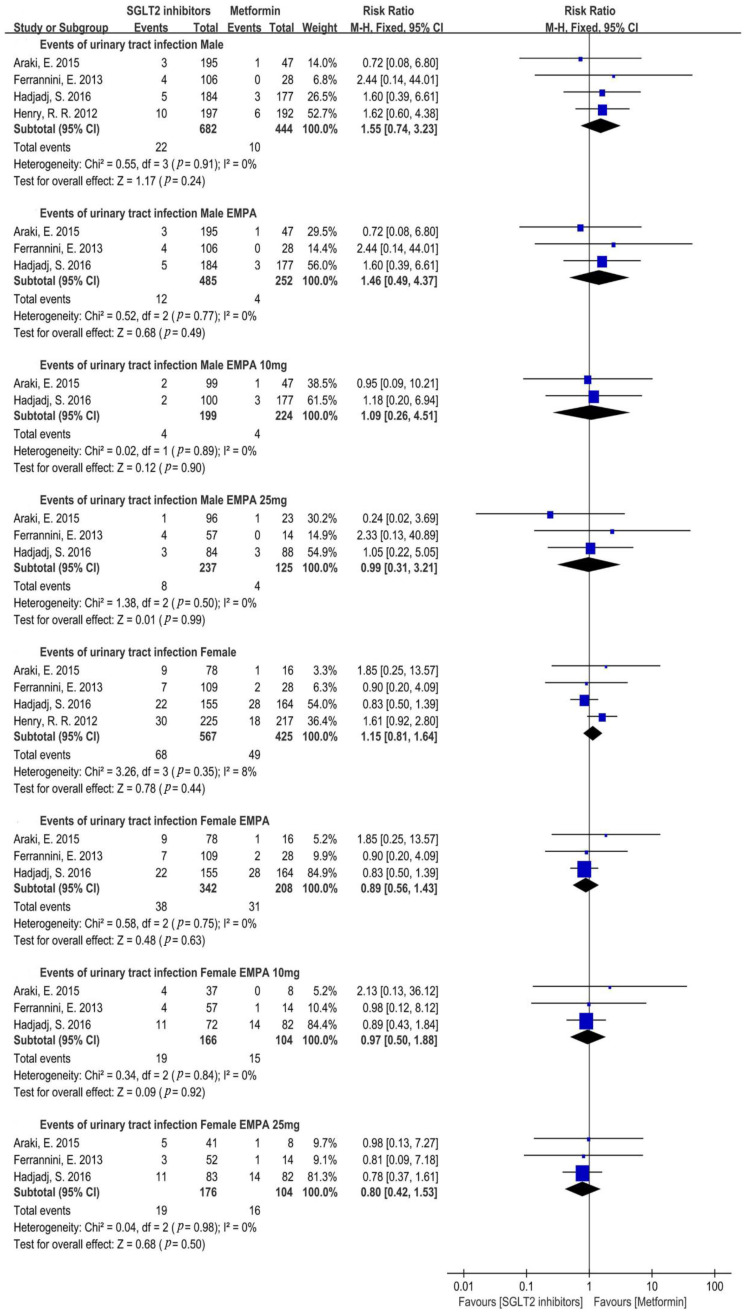
Risk of urinary tract infections with different types and doses of SGLT2i compared with that of metformin in male and female patients [20,22,23,24]. EMPA = empagliflozin.

**Figure 8:**
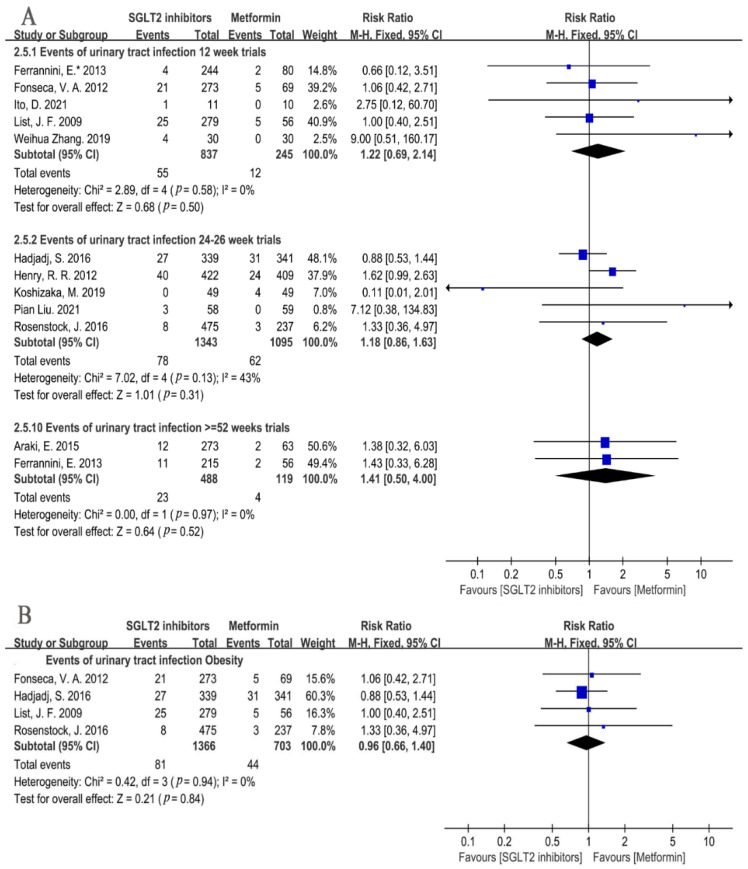
(**A**) Risk of urinary tract infections in patients with different treatment times of SGLT2i at 12 weeks, 24–26 weeks, and ≥52 weeks compared with that of metformin [20,21,22,23,24,25,26,27,28,29,31,32]. (**B**). Risk of urinary tract infections with SGLT2i compared with of metformin in obesity patients [22,25,29,31]. Obesity = SGLT2i used in obese people.

As for UTIs in different genders, a sex-specific subgroup was performed in five studies with the available gender data. The results showed that no significant differences were found in a UTI incidence in the SGLT2i versus the metformin group (Male: RR = 1.55, 95%CI = 0.74 to 3.23, *p* = 0.24, I^2^ = 0%, N = 4; Female: RR = 1.15, 95%CI = 0.81 to 1.64, *p* = 0.44, I^2^ = 8%, N = 4) (Figure 7). A further subgroup analysis demonstrated that empagliflozin had a similar risk of a UTI with metformin in males and females, and 10 mg and 25 mg of empagliflozin also had similar risk of a UTI compared to metformin (Male empagliflozin: RR = 1.46, 95%CI = 0.49 to 4.37, *p* = 0.49, I^2^ = 0%, N = 3; Male empagliflozin 10 mg: RR = 1.09, 95%CI = 0.26 to 4.51, *p* = 0.90, I^2^ = 0%, N = 2; Male empagliflozin 25 mg: RR = 0.99, 95%CI = 0.31 to 3.21, *p* = 0.99, I^2^ = 0%, N = 3; Female empagliflozin: RR = 0.89, 95%CI = 0.56 to 1.43, *p* = 0.63, I^2^ = 0%, N = 3; Female empagliflozin 10 mg: RR = 0.97, 95%CI = 0.50 to 1.88, *p* = 0.92, I^2^ = 0%, N = 3; Female empagliflozin 25 mg: RR = 0.80, 95%CI = 0.42 to 1.53, *p* = 0.50, I^2^ = 0%, N = 3) (Figure 7). Subgroup analysis of different treatment periods showed no significant difference in a UTI incidence between the SGLT2i and metformin group, no matter if the treatment was 12 weeks, 24–26 weeks, or ≥52 weeks (12 weeks: RR = 1.22, 95%CI = 0.69 to 2.14, *p* = 0.50, I^2^ = 0%, N = 5; 24–26 weeks: RR = 1.18, 95%CI = 0.86 to 1.63, *p* = 0.31, I^2^ = 43%, N = 5; ≥52 weeks: RR = 1.41, 95%CI = 0.50 to 4.00, *p* = 0.52, I^2^ = 0%, N = 2) (Figure 8A). 

It was noted that the obesity population accounted for the most T2DM patients. In our study, four trials enrolled the obese T2DM patients. Research indicates that obese people are more likely than people of a normal weight to develop infections of various types [33]. Surprisingly, we found that SGLT2i did not result in a higher risk of a UTI compared to the metformin in the obesity subgroup (RR = 0.96, 95%CI = 0.66 to 1.40, *p* = 0.84, I^2^ = 0%, N = 4) (Figure 8B).

Reproductive Tract Infections (RTI)

**Figure 9:**
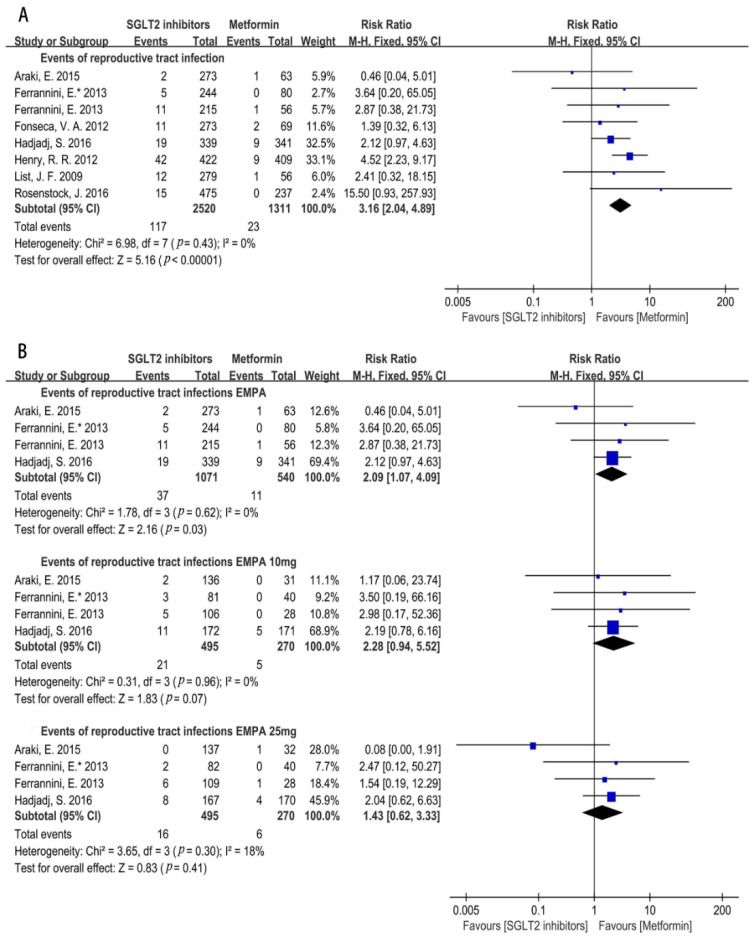
(**A**). Risk of reproductive tract infections in SGLT2i group compared with that of metformin [20,21,22,23,24,25,29,31]. (**B**). Risk of reproductive tract infections with EMPA and its different doses compared with that of metformin [20,21,22,23]. EMPA = empagliflozin.

For another type of genitourinary tract infections, an RTI, we also performed the corresponding analysis. The result showed that SGLT2i significantly increased the incidence of an RTI compared with that of metformin (RR = 3.16, 95%CI = 2.04 to 4.89, *p* < 0.00001, I^2^ = 0%, N = 8) (Figure 9A), and the incidence of an RTI induced by empagliflozin was also higher than that of metformin (RR = 2.09, 95%CI = 1.07 to 4.09, *p* = 0.03, I^2^ = 0%, N = 4) (Figure 9B). However, different doses of empagliflozin did not increase the risk of an RTI (empagliflozin 10 mg: RR = 2.28, 95%CI = 0.94 to 5.52, *p* = 0.07, I^2^ = 0%, N = 4; empagliflozin 25 mg: RR = 1.43, 95%CI = 0.62 to 3.33, *p* = 0.41, I^2^ = 18%, N = 4) (Figure 9B). The risk analysis of an RTI for dapagliflozin, canagliflozin, and ipragliflozin was not performed due to the lack of included literature.

**Figure 10:**
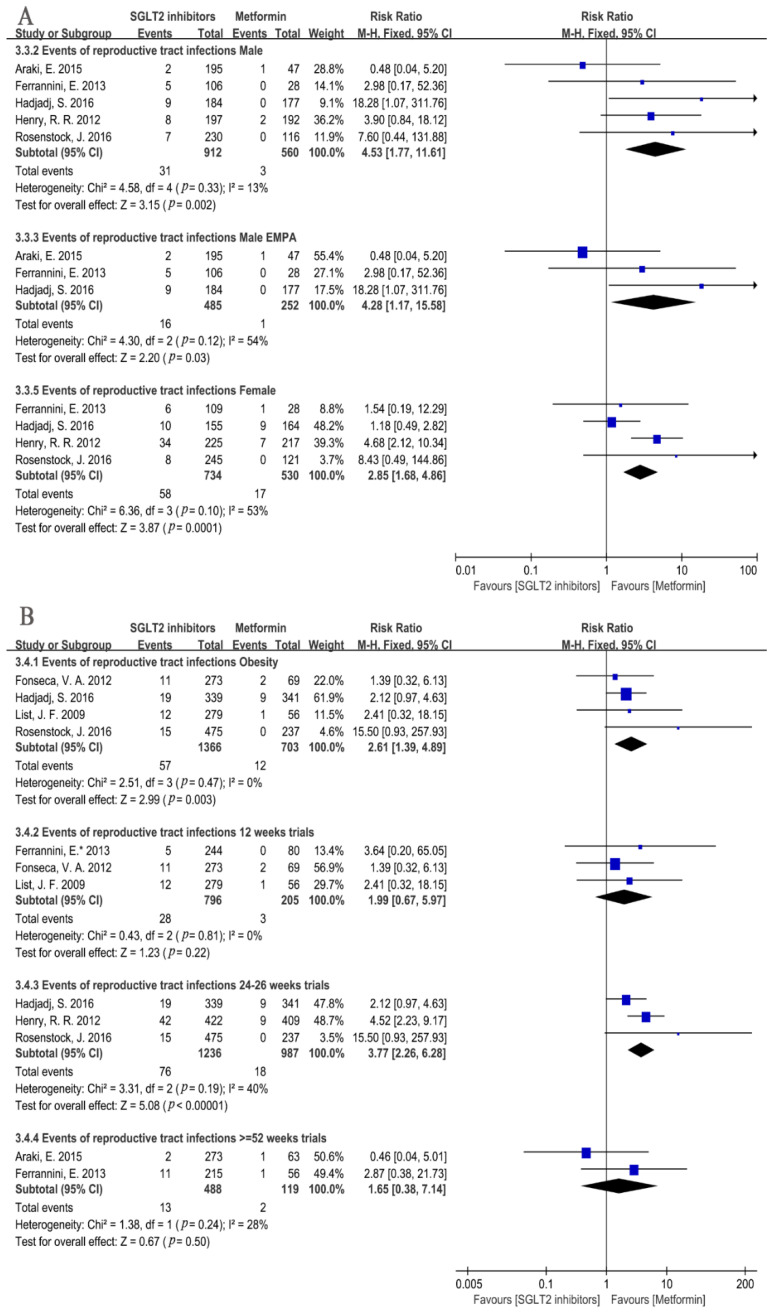
(**A**). Risk of reproductive tract infections with different types and doses of SGLT2i compared with that of metformin in male or female patients [20,22,23,24,29]. (**B**). Risk of reproductive tract infections in SGLT2i group compared with that metformin in obesity patients and risk of reproductive tract infections in patients with different treatment times of SGLT2i at 12 weeks or 24–26 weeks compared with that metformin [20,21,22,23,24,25,29,31]. EMPA = empagliflozin.

As for the sex-specific subgroup of RTIs, the results found that SGLT2i led to a higher incidence of an RTI for both male and female subgroups (Male: RR = 4.53, 95%CI = 1.77 to 11.61, *p* = 0.002, I^2^ = 13%, N = 5; Female: RR = 2.85, 95%CI = 1.68 to 4.86, *p* = 0.0001, I^2^ = 53%, N = 4, Figure 10A), which was in accordance with the aforementioned results (Figure 9A). In addition, treatment with empagliflozin resulted in a higher risk of an RTI in male patients (empagliflozin: RR = 4.28, 95%CI = 1.17 to 15.58, *p* = 0.03, I^2^ = 54%, N = 3) (Figure 10A).

Furthermore, SGLT2i elevated the incidence of an RTI in the obesity patients (RR = 2.61, 95%CI = 1.39 to 4.89, *p* = 0.003, I^2^ = 0%, N = 4) (Figure 10B). Additionally, for the time with SGLT2i therapy, the results revealed that SGLT2i treatment for 12 weeks showed a similar incidence of an RTI with that of metformin, whereas a significant increase in the incidence of an RTI in patients on SGLT2i treatment for 24–26 weeks were observed in contrast to the metformin treatment (12 weeks: RR = 1.99, 95%CI = 0.67 to 5.97, *p* = 0.22, I^2^ = 0%, N = 3; 24–26 weeks: RR = 3.77, 95%CI = 2.26 to 6.28, *p* < 0.00001, I^2^ = 40%, N = 3) (Figure 10B). Surprisingly, when using SGLT2i for more than 52 weeks, the increase in the incidence of an RTI did not quite reach a statistical significance (RR =1.65, 95%CI = 0.38 to 7.14, *p* = 0.50, I^2^ = 28%, N = 2) (Figure 10B).

Non-Genitourinary Tract Infections

**Figure 11:**
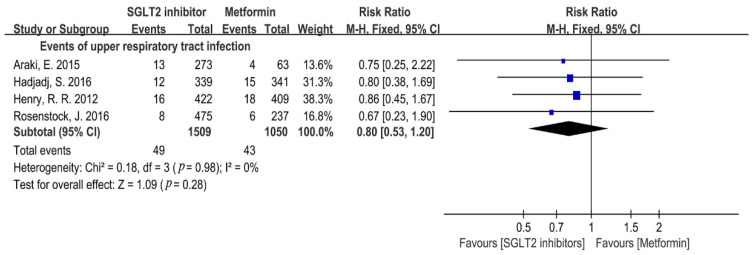
Risk of upper respiratory tract infections in SGLT2i group compared with that of metformin [20,22,24,29].

Four studies contributed to the risk of upper respiratory tract infections, and the result indicated no significant difference between the SGLT2i and metformin monotherapy group (RR = 0.80, 95%CI = 0.53 to 1.20, *p* = 0.28, I^2^ = 0%, N = 4) (Figure 11). In terms of skin infections, the occurrence of cellulitis was reported by three studies: two cases received 25 mg of empagliflozin, one case was with 5 mg of dapagliflozin, and one was with metformin. Henry, R. R. et al. reported one case of gangrene in the dapagliflozin 5 mg group. One case of herpes simplex was reported in the metformin group. Two cases of pulmonary infections were reported in the dapagliflozin 10 mg group. Additionally, one case of lobar Pneumonia was reported in the canagliflozin 100 mg group. Separate data describing the effects of SGLT2i on the infection types mentioned above were too few to draw reliable conclusions. 

#### 3.4.2. Effects on Cardiovascular Risk Factors

**Figure 12:**
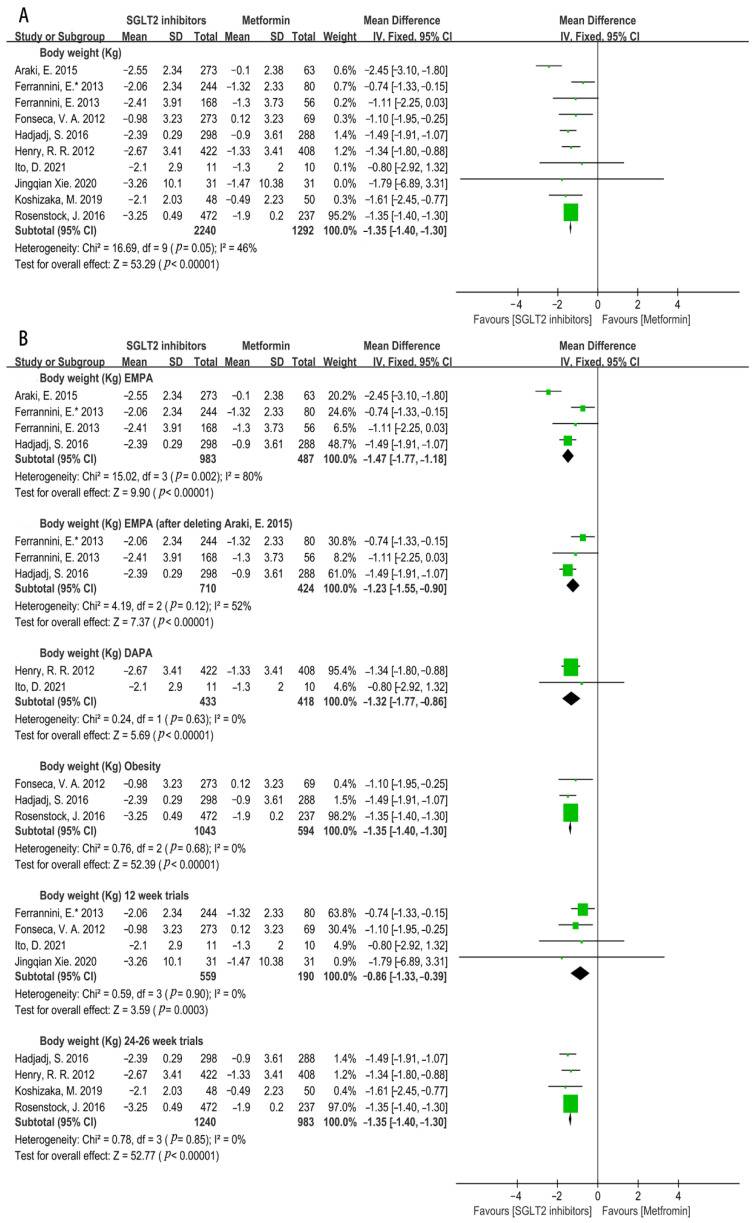
(**A**). Weighted mean difference in change in body weight (Kg) from baseline from SGLT2i compared with metformin [20,21,22,23,24,26,29,30,31,32]. (**B**). Weighted mean difference in change in body weight (Kg) from baseline from types of SGLT2i compared with metformin. Weighted mean difference in change in body weight (Kg) from baseline from SGLT2i used 12 weeks or 24–26 weeks compared with metformin and weighted mean difference in change in body weight (Kg) from baseline from SGLT2i in obesity patients [20,21,22,23,24,26,29,30,31,32]. DAPA = dapagliflozin, EMPA = empagliflozin. 12 weeks trials = SGLT2i used 12 weeks, 24–26 weeks trials = SGLT2i used 24–26 weeks. Obesity = SGLT2i used in obese people.

Obesity is one of the major causes of cardiovascular events [34]. Our meta-analysis showed the SGLT2i significantly reduced body weight compared with that of metformin (WMD = −1.35, 95%CI = −1.40 to −1.30, *p* < 0.00001, N = 10) with a moderate heterogeneity (I^2^ = 46%, Q test: *p* = 0.05) (Figure 12A). Therefore, a subgroup analysis was performed and found that dapagliflozin and empagliflozin notably reduced body weight compared with that of metformin (empagliflozin: WMD = −1.47, 95%CI = −1.77 to −1.18, *p* < 0.00001, I^2^ = 80%, N = 4; dapagliflozin: WMD = −1.32, 95%CI = −1.77 to −0.86, *p* < 0.00001, I^2^ = 0%, N = 2) (Figure 12B). Due to the high heterogeneity of the empagliflozin subgroup, a sensitivity analysis was conducted and the result revealed that the source of heterogeneity mainly came from the study of Araki, E. in 2015, in which sulfonylurea was added as background therapy. The sensitivity analysis suggested that empagliflozin showed more efficacy on the reduction in body weight than that of metformin with decreased heterogeneity (WMD = −1.23, 95%CI = −1.55 to −0.90, *p* < 0.00001, I^2^ = 52%, N = 3) (Figure 12B). Additionally, SGLT2i displayed a more significant decrease in body weight than that of metformin in obese T2DM patients (WMD = −1.35; 95%CI = −1.40 to −1.30; *p* < 0.00001, I^2^ = 0%, N = 3) (Figure 12B). Additionally, SGLT2i was superior to metformin in weight loss after treatment for both 12 and 24–26 weeks (12 weeks: WMD = −0.86, 95%CI = −1.33 to −0.39, *p* = 0.0003, I^2^ = 0%, N = 4; 24–26 weeks: WMD = −1.35, 95%CI = −1.40 to −1.30, *p* < 0.00001, I^2^ = 0%, N = 4) (Figure 12B). 

**Figure 13:**
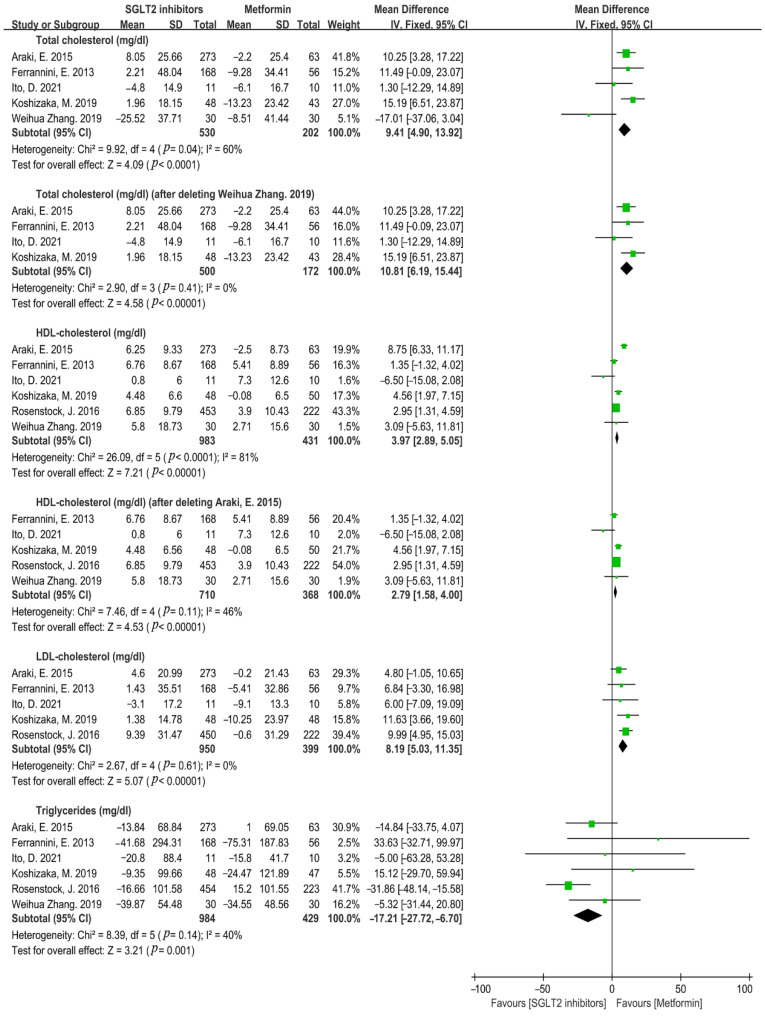
Weighted mean difference in change in blood lipid (mg/dL) from baseline from SGLT2i compared with metformin [20,23,26,28,29,32].

It is noted that hyperlipidemia is closely associated with cardiovascular disease [35]. SGLT2i presented an inferior effect on lowering the total cholesterol level compared to metformin with a high heterogeneity (WMD = 9.41, 95%CI= 4.90 to 13.92, *p* < 0.0001, I^2^ = 60%, N = 5), and the sensitivity analysis showed the same result after removing the study of Weihua Zhang in 2019, in which T2DM patients with metabolic syndrome were included (WMD = 10.81, 95%CI = 6.19 to 15.44, *p* < 0.00001, I^2^ = 0%, N = 4) (Figure 13). SGLT2i significantly increased HDL-cholesterol level compared to that of metformin with a strong heterogeneity (WMD = 3.97, 95%CI = 2.89 to 5.05, *p* < 0.00001, I^2^ = 81%, N = 6). Further sensitivity analysis showed that SGLT2i obviously upregulated the level of HDL-cholesterol after removing the study of Araki, E. in 2015, in which sulfonylurea was used as a background therapy with a reduced heterogeneity (WMD = 2.79, 95%CI = 1.58 to 4.00, *p* < 0.00001, I^2^ = 46%, N = 5). Compared with metformin, SGLT2i manifested less influence on LDL-cholesterol reduction, but significantly reduced the triglyceride level in contrast to metformin monotherapy (LDL-cholesterol: WMD = 8.19, 95%CI = 5.03 to 11.35, *p* < 0.00001, I^2^ = 0%, N = 5; triglyceride: WMD = −17.21, 95%CI = −27.72 to −6.70, *p* = 0.001, I^2^ = 40%, N = 6) (Figure 13).

**Figure 14:**
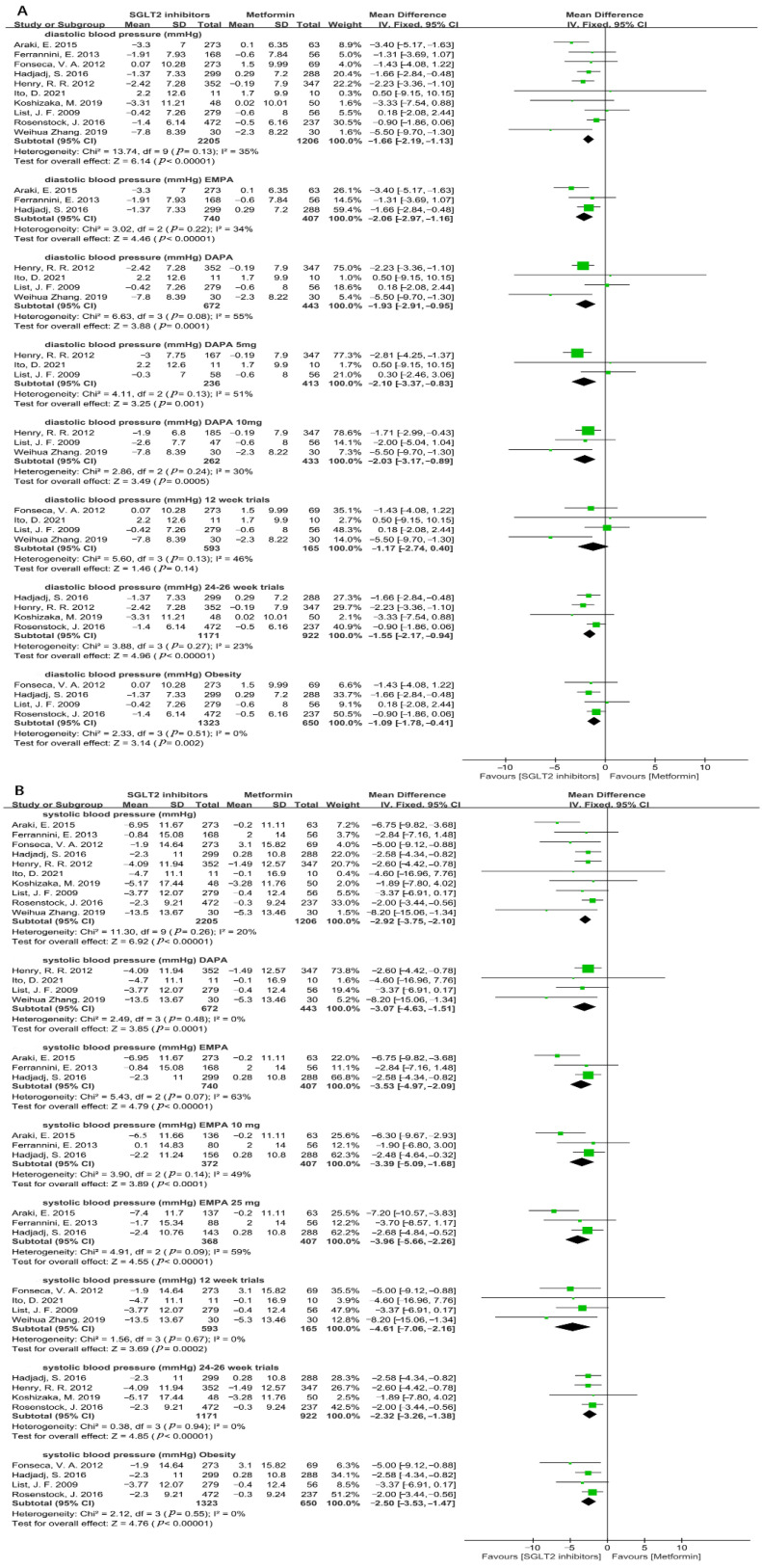
(**A**)**.** Weighted mean difference in change in diastolic blood pressure (mmHg) from baseline from SGLT2i compared with metformin [20,22,23,24,25,26,28,29,31,32]. (**B**). Weighted mean difference in change in systolic blood pressure (mmHg) from baseline from SGLT2i compared with metformin [20,22,23,24,25,26,28,29,31,32]. DAPA = dapagliflozin, EMPA = empagliflozin. 12 weeks trials = SGLT2i used 12 weeks, 24–26 weeks trials = SGLT2i used 24–26 weeks. Obesity = SGLT2i used in obese people.

Hypertension is an important risk factor of CVD [36]. Our results showed that there was a significant reduction in diastolic blood pressure (DBP) in the SGLT2i group versus the metformin group (WMD = −1.66, 95%CI = −2.19 to −1.13, *p* < 0.00001, I^2^ = 35%, N = 10) (Figure 14A). Among different SGLT2i, empagliflozin and dapagliflozin showed the stronger efficacy in lowering DBP than that of metformin with a moderate heterogeneity (empagliflozin: WMD = −2.06, 95%CI = −2.97 to −1.16, *p* < 0.00001, I^2^ = 34%, N = 3; dapagliflozin: WMD = −1.93, 95%CI = −2.91 to −0.95, *p* = 0.0001, I^2^ = 55%, N = 4) (Figure 14A). A subgroup analysis based on the dose of dapagliflozin was conducted, and the results demonstrated that both 5 mg and 10 mg of dapagliflozin could significantly decrease DBP (dapagliflozin5 mg: WMD= −2.10, 95%CI = −3.37 to −0.83, *p* = 0.001, I^2^ = 51%, N = 3; dapagliflozin 10 mg: WMD = −2.03, 95%CI = −3.17 to −0.89, *p* = 0.0005, I^2^ = 30%, N = 3) (Figure 14A). Additionally, there was a similar effect in reducing DBP between SGLT2i treatment for 12 weeks and metformin (WMD = −1.17, 95%CI = −2.74 to 0.40, *p* = 0.14, I^2^ = 46%, N = 4) (Figure 14A). Nonetheless, SGLT2i treatment for 24–26 weeks distinctly reduced DBP compared with that of metformin (WMD = −1.55, 95%CI = −2.17 to −0.94, *p* < 0.00001, I^2^ = 23%, N = 4) (Figure 14A). Four studies provided the DBP levels in obesity patients and the results indicated that SGLT2i decreased DBP more remarkably than metformin (WMD = −1.09, 95%CI = −1.78 to −0.41, *p* = 0.002, I^2^ = 0%) (Figure 14A).

In consistent with the result of DBP, SGLT2i significantly reduced systolic blood pressure (SBP) compared with metformin (WMD = −2.92, 95%CI = −3.75 to −2.10, *p* < 0.00001, I^2^ = 20%, N = 10) (Figure 14B). Likewise, the application of empagliflozin and dapagliflozin was more effective in reducing SBP than metformin (dapagliflozin: WMD = −3.07, 95%CI = −4.63 to −1.51, *p* = 0.0001, I^2^ = 0%, N = 4; empagliflozin: WMD = −3.53, 95%CI = −4.97 to −2.09, *p* < 0.00001, I^2^ = 63%, N = 3) (Figure 14B). Due to the non-negligible heterogeneity, a subgroup analysis based on the dose of empagliflozin was performed and the result revealed that both 10 mg and 25 mg of empagliflozin significantly reduced SBP compared with that of metformin (empagliflozin 10 mg: WMD = −3.39, 95%CI = −5.09 to −1.68, *p* < 0.0001, I^2^ = 49%, N = 3; empagliflozin 25 mg: WMD = −3.96, 95%CI = −5.66 to −2.26, *p* < 0.00001, I^2^ = 59%, N = 3) (Figure 14B). Considering the effect of various treatment periods of SGLT2i, we found that both SGLT2i treatment for 12 weeks and 24–26 weeks were associated with an obvious reduction in SBP compared with metformin (12 weeks: WMD = −4.61, 95%CI = −7.06 to −2.16, *p* = 0.0002, I^2^ = 0%, N = 4; 24–26 weeks: WMD = −2.32, 95%CI = −3.26 to −1.38, *p* < 0.00001, I^2^ = 0%, N = 4) (Figure 14B). In addition, for obese T2DM patients, treatment with SGLT2i significantly decreased SBP in contrast with metformin (WMD = −2.50, 95%CI = −3.53 to −1.47, *p* < 0.00001, I^2^ = 0%, N = 4) (Figure 14B).

#### 3.4.3. Efficacy on Glycemic Control 

**Figure 15:**
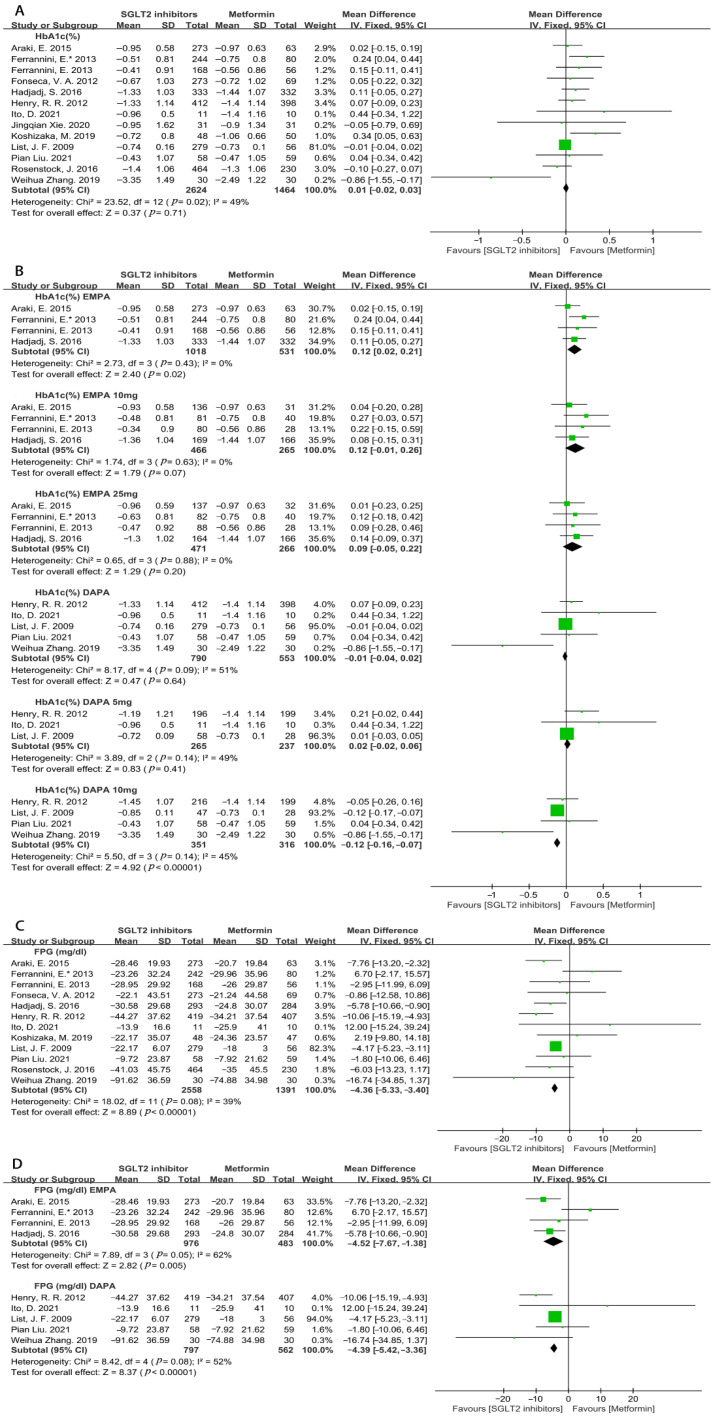
(**A**). Weighted mean differences in change in HbA1c (%) from baseline between SGLT2i and metformin [20,21,22,23,24,25,26,27,28,29,30,31,32]. (**B**). Weighted mean difference in change in HbA1c (%) from baseline from different types and doses of SGLT2i compared with metformin [20,21,22,23,24,25,26,27,28]. (**C**). Weighted mean difference in change in FPG (mg/dL) from baseline from SGLT2i compared with metformin [20,21,22,23,24,25,26,27,28,29,31,32]. (**D**). Weighted mean difference in change in FPG (mg/dL) from baseline from different types of SGLT2i compared with metformin [20,21,22,23,24,25,26,27,28]. DAPA = dapagliflozin, EMPA = empagliflozin.

The antihyperglycemic effects were evaluated and the results showed that SGLT2i and metformin had a similar effect on the reduction in HbA1c (WMD = 0.01, 95%CI = −0.02 to 0.03, *p* = 0.71, I^2^= 49%, N = 13) (Figure 15A). The subgroup analysis of empagliflozin displayed a slightly poor HbA1c reduction versus metformin (WMD = 0.12, 95%CI = 0.02 to 0.21, *p* = 0.02, I^2^ = 0%, N = 4) (Figure 15B). However, the doses subgroup analysis of empagliflozin showed no statistical difference between the SGLT2i and metformin groups (empagliflozin 10 mg: WMD = 0.12, 95%CI = −0.01 to 0.26, *p* = 0.07, I^2^ = 0%, N = 4; empagliflozin 25 mg: WMD = 0.09, 95%CI = −0.05 to 0.22, *p* = 0.20, I^2^ = 0%, N = 4) (Figure 15B). In addition, dapagliflozin showed the similar effect on lowering HbA1c compared with metformin (WMD = −0.01, 95%CI = −0.04 to 0.02, *p* = 0.64, I^2^ = 51%, N = 5) (Figure 15B). A further dose subgroup analysis found that 10 mg of dapagliflozin significantly reduced HbA1c, and 5 mg of dapagliflozin did not show a significant difference (dapagliflozin 5 mg: WMD = 0.02, 95%CI = −0.02 to 0.06, *p* = 0.41, I^2^ = 49%, N = 3; dapagliflozin 10 mg: WMD = −0.12, 95%CI = −0.16 to −0.07, *p* < 0.00001, I^2^ = 45%, N = 4) (Figure 15B). 

Compared to metformin monotherapy, SGLT2i significantly decreased FPG (WMD = −4.36, 95%CI = −5.33 to −3.40, *p* < 0.00001, I^2^ = 39%, N = 12) (Figure 15C). The subgroup analysis indicated that empagliflozin and dapagliflozin were more effective than metformin in the reduction in FPG (empagliflozin: WMD = −4.52, 95%CI = −7.67 to −1.38, *p* = 0.005, I^2^ = 62%, N = 4; dapagliflozin: WMD = −4.39, 95%CI = −5.42 to −3.36, *p* < 0.00001, I^2^ = 52%, N = 5) (Figure 15D).

#### 3.4.4. Hypoglycemia Incidence Risk

**Figure 16:**
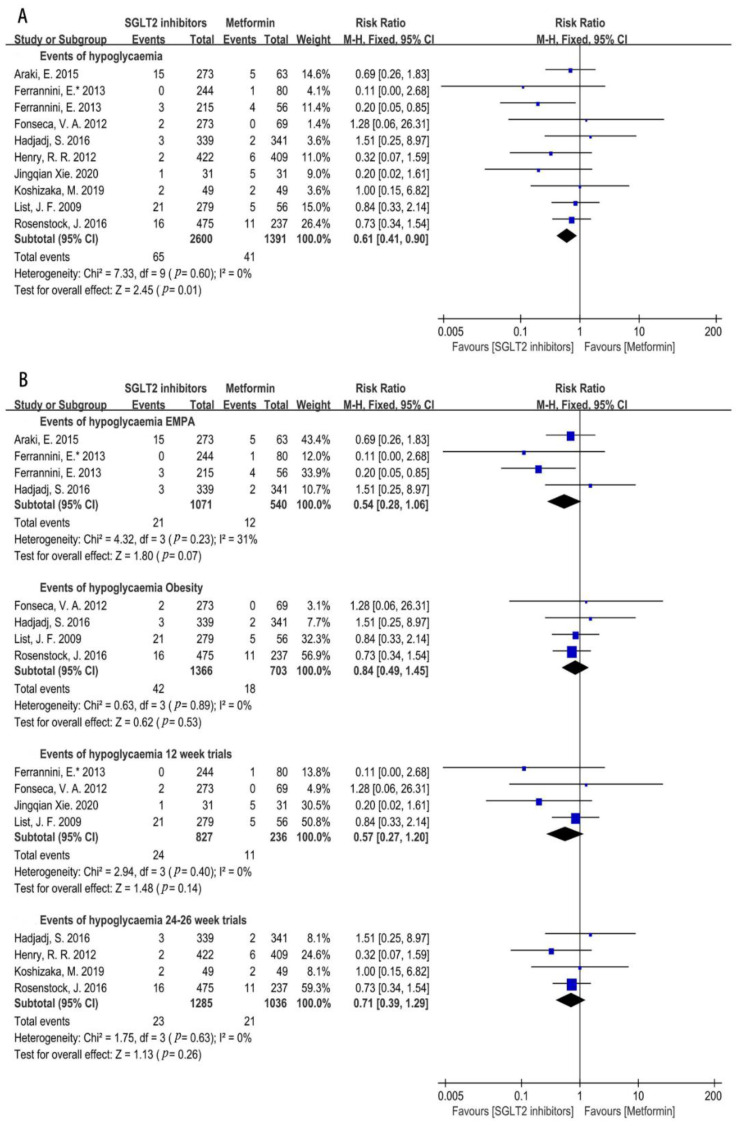
(**A**). Risk of hypoglycemia in SGLT2i group compared with that of metformin [20,21,22,23,24,25,29,30,31,32]. (**B**) Risk of hypoglycemia between the two groups in different subgroups [20,21,22,23,24,25,29,30,31,32]. EMPA = empagliflozin, 12 weeks trials = SGLT2i used 12 weeks, 24–26 weeks trials = SGLT2i used 24–26 weeks. Obesity = SGLT2i used in obese people.

Incidence of hypoglycemia was lower in SGLT2i groups compared with metformin, which was assessed from the ten trials included (RR = 0.61, 95%CI = 0.41 to 0.90, *p* = 0.01, I^2^ = 0%) (Figure 16A). A further subgroup analysis showed that the risk of hypoglycemia in the empagliflozin group was similar with that of metformin (RR = 0.54, 95%CI = 0.28 to 1.06, *p* = 0.07, I^2^ = 31%, N = 4) (Figure 16B). SGLT2i also showed a similar effect as metformin in the risk of hypoglycemia in obese people (RR = 0.84, 95%CI = 0.49 to 1.45, *p* = 0.53, I^2^ = 0%, N = 4) (Figure 16B). As for the influence of the treatment period of SGLT2i, both 12 weeks and 24–26 weeks of treatment of SGLT2i did not increase the incidence of hypoglycemia compared with metformin (12 weeks: RR = 0.57, 95%CI = 0.27 to 1.20, *p* = 0.14, I^2^ = 0%, N = 4; 24–26 weeks: RR = 0.71, 95%CI = 0.39 to 1.29, *p* = 0.26, I^2^ = 0%, N = 4) (Figure 16B). 

#### 3.4.5. Publication Bias

**Figure 17:**
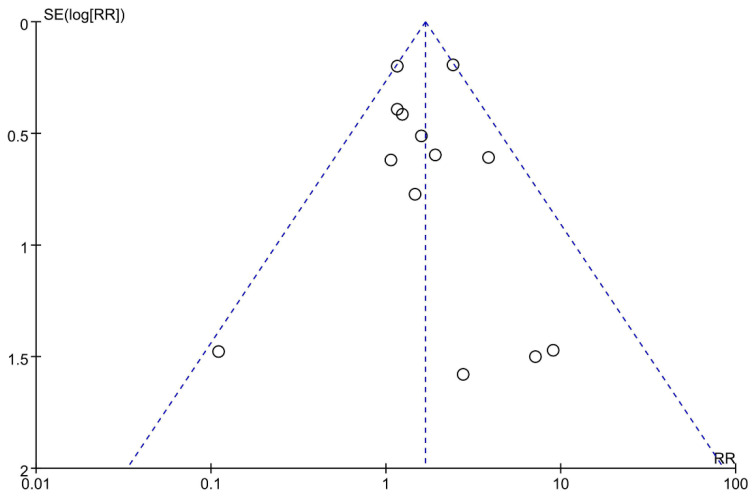
Funnel plot of genitourinary tract infection events.

Funnel plot of genitourinary tract infection events indicated no obvious reporting bias (Figure 17). Additionally, the Egger test also revealed no significant publication bias (*p* = 0.92).

## 4. Discussion

The prevalence of T2DM is increasing significantly in China, and oral glycemic agent therapy is still the main treatment strategy for most T2DM patients [37,38]. However, with a gradual loss of efficacy owing to the progressive loss of the β-cell function and the occurrence of metabolic comorbidities such as a gain in weight, CVD, and hypoglycemia, novel medications are being developed to improve the glycemic control and delay complications [39,40]. Sodium-glucose cotransporter 2 (SGLT2) mediates approximately 80–90% of renal glucose reabsorption under normal physiologic conditions, and SGLT2i can reduce 30–50% of the filtered glucose load in T2DM patients by increasing urinary glucose excretion [41]. However, the Food and Drug Administration (FDA) issued safety warnings for UTIs which may represent a leading cause of sepsis and are potentially life-threatening [42,43]. We conducted a meta-analysis to evaluate the benefit on the cardiovascular system and the potential risk of infection of SGLT2i in T2DM patients compared to metformin. Our analysis revealed strong associations between SGLT2i and the risk of genitourinary tract infections compared to metformin, and an RTI was the main cause of the difference, while there was no significant association between SGLT2i and the risk of UTI compared to metformin. This finding of our analysis is consistent with the results of previous reports, despite different trial designs and interventions of included studies from previous meta-analyses [44,45,46]. SGLT2i-induced increased glycosuria may predispose to mycotic colonization and bacterial overgrowth in view of the mechanism of action of the SGLT2i, in which case the enhanced risk of genitourinary tract infections is inevitable [47]. However, diuresis induced by SGLT2i may reduce urethra pathogen loads, which may be the reason for the non-obvious risk of a UTI [48,49]. 

Furthermore, the subgroup analysis of different types of SGLT2i indicated that empagliflozin was not associated with an increased risk of genitourinary tract infections, and a similar finding was observed in the risk of UTIs with empagliflozin. However, the increased risk of an RTI from the empagliflozin treatment was observed. Further analysis was conducted to separate the empagliflozin treatment into different dose groups, and we found neither 10 mg of empagliflozin nor 25 mg of empagliflozin increased the risk of an RTI compared to metformin. Dapagliflozin, the most commonly used SGLT2i in China, significantly increased the risk of genitourinary tract infections compared to metformin, potentially in a linearly dose-related mode. An animal study showed dapagliflozin has a more prolonged urinary glucose excretion than other SGLT2i [50]. Due to the lack of reasonable biologic mechanisms to explain this phenomenon, further research is required to decipher this finding. Canagliflozin was also associated with an increased risk of genitourinary tract infections, whereas ipragliflozin did not and the conclusions should be treated cautiously due to the small number of RCTs that reported this outcome.

It is uncertain whether there is a sex difference in the risk of UTIs and RTIs associated with SGLT2i compared to metformin because of the different physiological structure of the urogenital tract. Our analysis revealed that SGLT2i was associated with a higher risk of an RTI in the male and female population compared with metformin. The conclusions are consistent with previous studies which reported that increased genital infections associated with SGLT2i were common in females and males [51,52,53]. In view of various treatment periods of SGLT2i, we conducted a further subgroup analysis using Revman software to clarify the effect of the treatment period of SGLT2i on the infection incidence. We found that SGLT2i therapy for 12 weeks did not increase the risk of UTIs and RTIs, and the incidence in an RTI was higher at 24 to 26 weeks than that of metformin, which was consistent with the previous studies [54,55,56]. Interestingly, the subgroup analysis showed there was no difference on the risk of RTIs and UTIs between SGLT2i and metformin at 52 weeks’ treatment. Due to the small number of articles included, more research is needed to confirm the long-term safety of SGLT2i on UTIs and RTIs.

T2DM works as a serious chronic metabolic disease that is characterized by insulin resistance (IR) and obesity, which accounts for 44% of diabetes cases [57,58]. Obesity is associated with the worsening of IR, and there is a 7-fold increased risk in the individuals’ mortality in T2DM with obese individuals [59]. Our research indicated that obese T2DM patients treated with SGLT2i suffered a higher risk of RTI compared to metformin monotherapy. Recent studies have proved that obesity could alter immune cell function and glucose metabolism, and these obesity-related mechanisms may contribute to the predisposition to infections [60]. Therefore, weight loss supported the reduction in infection risk. 

With the exception of the most common genitourinary tract infections, sporadic studies have reported that SGLT2i was associated with skin and pulmonary infections [61,62,63]. Urticaria, pruritus, photosensitivity, and various rashes caused by SGLT2i may be driven by a potential skin toxicity [61,62]. In addition, SGLT2i also led to an increased liquid glucose concentration of the airway surface, which translated directly to an increased proliferation of pathogenic bacteria and induced the occurrence of lung infections, for some SGLT2i bound with the SGLT-1 receptors in the lungs, such as canagliflozin and ipragliflozin [63,64]. Hence, we conducted further research to determine whether SGLT2i increased the risk of a potential extra-urogenital infection. Our research showed that SGLT2i treatment was not significantly associated with an increased risk of upper respiratory tract infections in contrast to metformin. However, due to the small number of RCTs that reported related data, further research is required to broaden the progress of this field. Additionally, rare studies focused on the influence of SGLT2i on the skin and other ectopic infections, therefore, no pooled data were analyzed to clarify the effect of SGLT2i on the cutaneous infection in our systematic review.

Due to the severe detriment and high mortality rate of diabetes-related cardiovascular complications, antidiabetic agents should be prioritized in T2DM patients complicated with cardiovascular disease, especially the drugs that could effectively improve cardiovascular outcomes [65,66,67]. Our findings strongly supported the use of SGLT2i in T2DM patients were related with the decreased risk of cardiovascular disease, and SGLT2i showed a potential beneficial effect on reducing cardiovascular risk factors when compared to metformin monotherapy. T2DM patients treated with SGLT2i experienced a significant body weight reduction, regardless of the type of SGLT2i, the treatment duration, and the baseline weight of the patients. The effect of SGLT2i on lowering body weight may be due to the glycosuria-mediated effect of osmotic diuresis and caloric expenditure [68,69]. Furthermore, as for the regulation of the lipid metabolism, SGLT2i could significantly enhance the level of HDL cholesterol and decrease triglyceride levels, which further lead to the decrease in cardiovascular risk. This may be explained by the fact that SGLT2i improves IR and increases endogenous insulin secretion, which in turn increases the catabolism of very low density lipoprotein (VLDL) cholesterol and chylomicrons, ultimately leading to a reduction in triglycerides and an increase in HDL cholesterol [70]. Similarly, the beneficial effect of SGLT2i on the modulation of blood pressure was also confirmed by our meta-analysis, which was consistent with the previous reports [71,72]. Osmotic diuresis caused by increasing urinary glucose excretion induced by SGLT2i is likely to be the reason for the antihypertensive effect [73]. To sum up, the previously described beneficial effect of SGLT2i on improving the risk of cardiovascular outcomes was confirmed by our systematic review [12,72]. 

From the view of the antihyperglycemic effect, our research suggested that SGLT2i monotherapy, which was developed for the treatment of T2DM, showed a similar improvement of HbA1c with metformin. Moreover, we found that 10 mg of dapagliflozin appeared to produce a slightly greater reduction in HbA1c than metformin for T2DM patients. For another indicator that effectively reflects the antihyperglycemic efficacy, a significant reduction in FPG in SGLT2i monotherapy was observed in comparison with metformin. Considering the potential risk of hypoglycemia, which is considered as the acute complication of diabetes and severely threatens the life of patients [74], we conducted the analysis of adverse event about the incidence of hypoglycemia. Our research indicated that SGLT2i displayed a low risk of hypoglycemia, which showed no significant difference in each subgroup analysis compared with metformin. 

Our meta-analysis firstly focused on the cardiovascular benefit and infection risk of SGLT2i compared with metformin, which filled many gaps left by those of prior work. In this analysis, we included more recent articles and focused on the comparison of the most relevant infection events and potential efficacy, including the reduction in the cardiovascular risk factor and improvement of glycemic control with the current first-line antihyperglycemic agent, metformin. Furthermore, limitations also existed in our research. First, the cardiovascular outcomes were not reported in the original trials, so the cardiovascular benefits in our study were evaluated by the risk factors. Second, the wide confidence intervals lead to a low level of certainty for some findings and need to be interpreted with care. In addition, few high-quality articles which reported the influence of canagliflozin and ipragliflozin were included, hence, more RCTs are needed to address the large evidence gap on the efficacy and safety of these SGLT2i.

## 5. Conclusions

In summary, SGLT2i showed significant benefits in the reduction in cardiovascular risk factors, such as body weight, blood pressure, triglycerides, and increasing HDL cholesterol level, and had similar antihyperglycemic efficacy, including lowering blood glucose and HbA1c without the significant elevation of UTIs compared with metformin monotherapy, especially in obese T2DM patients. In short-term trials, SGLT2i provided the similar antihyperglycemic effect with metformin and induced additional cardiovascular benefits and the potential risk of an RTI. Additional long-term trials are needed to confirm the long-term safety of SGLT2i, which is expected to be the first choice for patients with metformin intolerance.

## Figures and Tables

**Table 1:** Basic characteristics of randomized controlled trials included in the meta-analysis.

Study	Interventions	Study Duration (Weeks)	Number of Participants	Male (N)	Age (Years)	HbA1c (%)	Body Weight (Kg)	BMI (Kg/M)
Araki, E. 2015, [20] (NCT01368081)	EMPA 10 mg	52	136	99	61.3 ± 9.9	8.0 ± 0.7	65.8 ± 12.2	24.6 ± 3.8
	EMPA 25 mg		137	96	61.8 ± 9.6	8.1 ± 0.8	67.0 ± 13.7	25.2 ± 4.2
	MET 1111 mg ^§^		63	47	60.0 ± 10.2	7.9 ± 0.8	68.2 ± 12.2	25.2 ± 3.6
Ferrannini, E.* 2013, [21] (NCT00789035)	EMPA 5 mg	12	81	46	59.0 (37–78) *	7.9 ± 0.8	82.8 (51.9–116.0) *	28.5 (20.5–38.8) *
	EMPA 10 mg		81	40	58.0 (30–76) *	8.0 ± 0.8	76.8 (45.5–118.0) *	28.1 (21.5–39.3) *
	EMPA 25 mg		82	41	57.0 (30–79) *	7.8 ± 0.8	81.2 (49.1–130.0) *	28.3 (20.1–38.8) *
	MET †		80	39	58.0 (34–73) *	8.1 ± 0.9	81.1 (42.0–126.0) *	28.6 (18.7–40.6) *
Hadjadj, S. 2016, [22] (NCT01719003)	EMPA 10 mg	24	169	97	53.1 ± 10.7	8.6 ± 1.2	83.8 ± 19.8	30.3 ± 5.2
	EMPA 25 mg		164	83	53.3 ± 10.7	8.9 ± 1.3	83.1 ± 20.3	30.6 ± 5.9
	MET 500 mg		168	86	53.4 ± 10.9	8.7 ± 1.0	82.7 ± 21.2	30.3 ± 5.8
	MET 1000 mg		164	92	51.6 ± 10.8	8.6 ± 1.1	83.7 ± 20.1	30.5 ± 5.9
Ferrannini, E. 2013, [23] (NCT00881530)	EMPA 10 mg	78	106	49	59 (30–76) *	7.9 ± 0.9	82.9 ± 16.4	28.9 (20.3–39.2) *
	EMPA 25 mg		109	57	59 (35–79) *	8.0 ± 0.9	84.6 ± 18.1	28.1 (19.3–40.0) *
	MET †		56	28	58 (35–73) *	8.2 ± 1.0	85.8 ± 15.6	28.6 (22.4–39.3) *
Henry, R.R. 2012, [24] (NCT00643851 NCT00859898)	DAPA 5 mg	24	203	92	52.3 ±10.2	9.1 ± 1.4	86.2 ± 21.1	NO
	DAPA 10 mg		219	105	51.1 ± 11.5	9.1 ± 1.3	88.5 ± 19.3	
	MET 2000 mg		409	192	52.3 ± 10.1	9.1 ± 1.3	86.4 ± 19.7	
List, J.F. 2009, [25] (NCT00263276)	DAPA 2.5 mg	12	59	29	55.0 ± 11.0	7.6 ± 0.7	90.0 ± 20.0	32.0 ± 5.0
	DAPA 5 mg		58	28	55.0 ± 12.0	8.0 ± 0.9	89.0 ± 17.0	32.0 ± 5.0
	DAPA 10 mg		47	25	54.0 ± 9.0	8.0 ± 0.8	86.0 ± 17.0	31.0 ± 5.0
	DAPA 20 mg		59	32	55.0 ± 10.0	7.7 ± 0.9	88.0 ± 18.0	31.0 ± 5.0
	DAPA 50 mg		56	25	53.0 ± 10.0	7.8 ± 1.0	92.0 ± 19.0	32.0 ± 4.0
	MET 1500 mg		56	27	54.0 ± 9.0	7.6 ± 0.8	88.0 ± 20.0	32.0 ± 5.0
Ito, D. 2021, [26]	DAPA 5 mg	12	11	8	55.9 ± 7.5	7.9 ± 0.9	77.5 ± 18.1	27.7 ± 4.9
	MET 1000 mg		10	9	57.5 ± 9.6	7.9 ± 0.9	74.8 ± 8.7	26.7 ± 3.4
Pian Liu. 2021, [27]	DAPA 10 mg	26	58	31	66.6 ± 8.4	8.1 ± 1.2	70.1 ± 7.8	24.7 ± 1.8
	MET 1000 mg		59	32	66.3 ± 9.3	8.5 ± 1.1	68.6 ± 7.7	24.1 ± 2.3
Weihua Zhang. 2019, [28]	DAPA 10 mg	12	30	19	44.9 ± 10.2	8.5 ± 1.7	76.3 ± 13.6	27.9 ± 4.3
	MET 1500 mg		30	20	44.1 ± 10.8	8.3 ± 1.4	75.4 ± 14.3	27.5 ± 4.5
Rosenstock, J. 2016, [29] (NCT01809327)	CANA 100 mg	26	237	105	54.1 ± 10.7	8.8 ± 1.2	90.2 ± 18.6	32.4 ± 5.4
	CANA 300 mg		238	125	55.9 ± 9.6	8.8 ± 1.2	93.0 ± 19.9	32.6 ± 5.8
	MET 2000 mg		237	116	55.3 ± 9.8	8.8 ± 1.2	92.1 ± 20.1	33.0 ± 6.0
Jingqian Xie. 2020, [30]	CANA 100 mg	12	31	11	63.8 ± 8.6	9.1 ± 1.7	73.3 ± 10.3	NO
	MET (1000–1500 mg)		31	13	63.0 ± 9.7	8.3 ± 1.5	72.5 ± 10.2	
Fonseca, V.A. 2012, [31] (NCT01071850)	IPRA 12.5 mg	12	70	39	53.9 ± 9.6	8.0 ± 0.8	86.0 ± 22.3	31.0 ± 5.9
	IPRA 50 mg		67	34	52.6 ± 10.7	8.1 ± 0.8	90.7 ± 20.8	32.2 ± 5.9
	IPRA 150 mg		68	29	54.2 ± 10.3	7.8 ± 0.7	83.3 ± 21.6	30.9 ± 6.3
	IPRA 300 mg		68	37	54.2 ± 10.7	7.9 ± 0.7	86.7 ± 19.6	30.7 ± 5.0
	MET 1500 mg		69	40	53.1 ± 11.7	8.0 ± 0.9	84.1 ± 21.8	29.8 ± 5.5
Koshizaka, M. 2019, [32]	IPRA 50 mg	12	48	31	56.6 ± 11.9	8.0 ± 0.7	73.1 ± 14.2	27.6 ± 4.2
	MET 1124 mg^§^		50	28	55.7 ± 12.2	8.1 ± 0.9	78.3 ± 18.4	28.8 ± 5.3

EMPA, empagliflozin; DAPA, dapagliflozin; CANA, canagliflozin; IPRA, ipragliflozin; MET, metformin; BMI, body mass index. Data are mean ± SD (standard deviation) unless indicated otherwise. Ferrannini, E.*: Used to distinguish two articles with the same first author name and publication year (references [21,23]). * Data are median (minimum–maximum).† MET dose < 1000 mg or up to the maximum tolerated dose. § Data are mean dose.

## Data Availability

Data are contained within the article or Supplementary Material.

## Supplementary Materials

The following supporting information can be downloaded at: https://www.mdpi.com/article/10.3390/metabo12100979/s1, Supplementary File S1. Search Strategy (DOC).
