# Supplementary material for: The Cardiovascular Benefits and Infections Risk of SGLT2i versus Metformin in Type 2 Diabetes: A Systemic Review and Meta-Analysis
Source: Metabolites. 2022 Oct 17;12(10):979. doi: 10.3390/metabo12100979 (PMC9610220; doi:10.3390/metabo12100979)
Supplement: Supplementary file 1 [file metabolites-12-00979-s001.zip › metabolites-1928216-Supplementary File S1.pdf]

Search Strategy

PUBMED Search Strategy：

#1: "Sodium-Glucose Transporter 2"[Mesh]

#2: "sodium glucose transporter 2"[Title/Abstract] OR "slc5a2 protein"[Title/Abstract] OR "sglt2 protein"[Title/Abstract] OR "SGLT2i"[Title/Abstract]

#3: "dapagliflozin"[Title/Abstract] OR "Farxiga"[Title/Abstract] OR "Forxiga"[Title/Abstract] OR "BMS 512148"[Title/Abstract] OR "BMS512148"[Title/Abstract] OR "BMS-512148"[Title/Abstract]

#4: "Empagliflozin"[Title/Abstract] OR "BI 10773"[Title/Abstract] OR "BI10773"[Title/Abstract] OR "BI-10773"[Title/Abstract] OR "Jardiance"[Title/Abstract]

#5: "Canagliflozin"[MeSH Terms] OR "Invokana"[Title/Abstract] OR "Canagliflozin Hemihydrate"[Title/Abstract]

#6: "ertugliflozin"[Title/Abstract] OR "Steglatro"[Title/Abstract] OR "PF 04971729"[Title/Abstract] OR "PF04971729"[Title/Abstract] OR "PF-04971729"[Title/Abstract]

#7: "ipragliflozin"[Title/Abstract] OR "Suglat"[Title/Abstract] OR "ASP1941"[Title/Abstract] OR "ASP-1941"[Title/Abstract]

#8: "luseogliflozin"[Title/Abstract] OR "Lusefi"[Title/Abstract] OR "TS 071"[Title/Abstract] OR "TS-071"[Title/Abstract]

#9: "CSG452"[Title/Abstract] OR "Apleway"[Title/Abstract] OR "Deberza"[Title/Abstract] OR "tofogliflozin"[Title/Abstract]

#10: "sotagliflozin"[Title/Abstract] OR "LX4211"[Title/Abstract] OR "LX-4211"[Title/Abstract] OR "zynquista"[Title/Abstract]

#11: "remogliflozin etabonate"[Title/Abstract]

#12: "sergliflozin etabonate"[Title/Abstract]

#13: #1 OR #2 OR #3 OR #4 OR #5 OR #6 OR #7 OR #8 OR #9 OR #10 OR #11 OR #12

#14: "Metformin"[MeSH Terms] OR "Dimethylbiguanidine"[Title/Abstract] OR "Dimethylguanylguanidine"[Title/Abstract] OR "Glucophage"[Title/Abstract] OR "Metformin Hydrochloride"[Title/Abstract] OR "hydrochloride metformin"[Title/Abstract] OR "Metformin HCl"[Title/Abstract]

#15: "Diabetes Mellitus"[MeSH Terms] OR "diabetes mellitus experimental"[Title/Abstract] OR "diabetes mellitus type 1"[Title/Abstract] OR "Wolfram Syndrome"[Title/Abstract] OR "diabetes mellitus type 2"[Title/Abstract] OR "diabetes mellitus lipoatrophic"[Title/Abstract] OR "diabetes gestational"[Title/Abstract] OR "Diabetic Ketoacidosis"[Title/Abstract] OR "Donohue Syndrome"[Title/Abstract] OR "Latent Autoimmune Diabetes in Adults"[Title/Abstract] OR "Prediabetic State"[Title/Abstract] OR "Diabetes Complications"[Title/Abstract] OR "Diabetic Angiopathies"[Title/Abstract] OR "Diabetic Cardiomyopathies"[Title/Abstract] OR "Diabetic Coma"[Title/Abstract] OR "Diabetic Nephropathies"[Title/Abstract] OR "Diabetic Neuropathies"[Title/Abstract] OR "Fetal Macrosomia"[Title/Abstract] OR "Diabetes"[Title/Abstract] OR "DM"[Title/Abstract]

#16: #13 AND #14 AND #15

EMBASE Search Strategy:

#1: 'sodium glucose cotransporter 2 inhibitor'/exp OR 'sodium glucose cotransporter 2 inhibitor'

#2: gliflozin:ti,ab,kw OR 'gliflozin derivative':ti,ab,kw OR gliflozins:ti,ab,kw OR 'sglt2 inhibitor':ti,ab,kw OR 'sglt2 inhibitors':ti,ab,kw OR 'sodium dependent glucose cotransporter 2 inhibitor':ti,ab,kw OR 'sodium glucose co-transporter 2 inhibitor':ti,ab,kw OR 'sodium-glucose transporter 2 inhibitors':ti,ab,kw

#3: #1 OR #2

#4: 'dapagliflozin'/exp OR 'dapagliflozin'

#5: '1 [4 chloro 3':ti,ab,kw AND '4 ethoxybenzyl':ti,ab,kw AND 'phenyl] 1 deoxy beta d glucopyranose':ti,ab,kw OR (2:ti,ab,kw AND 3:ti,ab,kw AND '4 ethoxybenzyl':ti,ab,kw AND '4 chlorophenyl':ti,ab,kw AND '6 hydroxymethyltetrahydro 2h pyran 3, 4, 5 triol':ti,ab,kw) OR ('2 [4 chloro 3':ti,ab,kw AND '4 ethoxybenzyl':ti,ab,kw AND 'phenyl] 6':ti,ab,kw AND hydroxymethyl:ti,ab,kw AND 'oxane 3, 4, 5 triol':ti,ab,kw) OR ('2 [4 chloro 3 [':ti,ab,kw AND '4 ethoxyphenyl':ti,ab,kw AND 'methyl] phenyl] 6':ti,ab,kw AND hydroxymethyl:ti,ab,kw AND 'oxane 3, 4, 5 triol':ti,ab,kw) OR 'bms 512148':ti,ab,kw OR 'bms512148':ti,ab,kw OR 'dapagliflozin acetate':ti,ab,kw OR 'dapagliflozin propanediol':ti,ab,kw OR 'dapagliflozin propanediol monohydrate':ti,ab,kw OR edistride:ti,ab,kw OR farxiga:ti,ab,kw OR forxiga:ti,ab,kw

#6: #4 OR #5

#7: 'empagliflozin'/exp OR 'empagliflozin'

#8: '1, 5 anhydro 1 [4 chloro 3 [4 [':ti,ab,kw AND 'tetrahydro 3 furyl':ti,ab,kw AND 'oxy] benzyl] phenyl] glucitol':ti,ab,kw OR ('1, 5 anhydro 1 [4 chloro 3 [[4 [':ti,ab,kw AND '3 oxolanyl':ti,ab,kw AND 'oxy] phenyl] methyl] phenyl] glucitol':ti,ab,kw) OR ('1, 5 anhydro 1 [4 chloro 3 [[4 [':ti,ab,kw AND 'tetrahydro 3 furanyl':ti,ab,kw AND 'oxy] phenyl] methyl] phenyl] glucitol':ti,ab,kw) OR ('1, 5 anhydro 1 c [4 chloro 3 [[4 [':ti,ab,kw AND 'oxolan 3 yl':ti,ab,kw AND 'oxy] phenyl] methyl] phenyl] dextro glucitol':ti,ab,kw) OR ('1, 5 anhydro 1 c [4 chloro 3 [[4 [':ti,ab,kw AND 'tetrahydro 3 furanyl':ti,ab,kw AND 'oxy] phenyl] methyl] phenyl] dextro glucitol':ti,ab,kw) OR 'bi 10773':ti,ab,kw OR 'bi10773':ti,ab,kw OR jardiance:ti,ab,kw

#9: #7 OR #8

#10: 'canagliflozin'/exp OR 'canagliflozin'

#11: '1, 5 anhydro 1 c [3 [5':ti,ab,kw AND '4 fluorophenyl':ti,ab,kw AND '2 thenyl] 4 methylphenyl] glucitol':ti,ab,kw OR ('1, 5 anhydro 1 c [3 [[5':ti,ab,kw AND '4 fluorophenyl':ti,ab,kw AND '2 thienyl] methyl] 4 methylphenyl] d glucitol':ti,ab,kw) OR ('1, 5 anhydro 1 c [3 [[5':ti,ab,kw AND '4 fluorophenyl':ti,ab,kw AND 'thiophen 2 yl] methyl] 4 methylphenyl] d glucitol':ti,ab,kw) OR ('2 [3 [5':ti,ab,kw AND '4 fluorophenyl':ti,ab,kw AND 'thiophen 2 ylmethyl] 4 methylphenyl] 6':ti,ab,kw AND hydroxymethyl:ti,ab,kw AND 'tetrahydropyran 3, 4, 5 triol':ti,ab,kw) OR canagliflocin:ti,ab,kw OR 'canagliflozin hemihydrate':ti,ab,kw OR invokana:ti,ab,kw OR 'jnj 28431754':ti,ab,kw OR 'jnj28431754':ti,ab,kw OR 'ta 7284':ti,ab,kw OR 'ta7284':ti,ab,kw

#12: #10 OR #11

#13: 'ertugliflozin'/exp OR 'ertugliflozin'

#14: '1, 6 anhydro 1 [4 chloro 3':ti,ab,kw AND '4 ethoxybenzyl':ti,ab,kw AND 'phenyl] 5 hydroxymethyl beta l idopyranose':ti,ab,kw OR ('1, 6 anhydro 1 c [4 chloro 3 [':ti,ab,kw AND '4 ethoxyphenyl':ti,ab,kw AND 'methyl] phenyl] 5 c':ti,ab,kw AND hydroxymethyl:ti,ab,kw AND 'beta l idopyranose':ti,ab,kw) OR ('5 [4 chloro 3':ti,ab,kw AND '4 ethoxybenzyl':ti,ab,kw AND 'phenyl] 1 hydroxymethyl 6, 8 dioxabicyclo [3.2.1] octane 2, 3, 4 triol':ti,ab,kw) OR ('5 [4 chloro 3 [':ti,ab,kw AND '4 ethoxyphenyl':ti,ab,kw AND 'methyl] phenyl] 1':ti,ab,kw AND hydroxymethyl:ti,ab,kw AND '6, 8 dioxabicyclo [3.2.1] octane 2, 3, 4 triol':ti,ab,kw) OR 'ertugliflozin pidolate':ti,ab,kw OR 'ertugliflozin pyroglutamic acid':ti,ab,kw OR 'mk 8835':ti,ab,kw OR mk8835:ti,ab,kw OR 'pf 04971729':ti,ab,kw OR 'pf 04971729 00':ti,ab,kw OR 'pf 04971729-00':ti,ab,kw OR 'pf 4971729':ti,ab,kw OR 'pf 4971729 00':ti,ab,kw OR 'pf 4971729-00':ti,ab,kw OR 'pf04971729':ti,ab,kw OR 'pf04971729 00':ti,ab,kw OR 'pf04971729-00':ti,ab,kw OR 'pf4971729':ti,ab,kw OR 'pf4971729 00':ti,ab,kw OR 'pf4971729-00':ti,ab,kw OR steglatro:ti,ab,kw

#15: #13 OR #14

#16: 'ipragliflozin'/exp OR 'ipragliflozin'

#17: '1, 5 anhydro 1 c [3 [':ti,ab,kw AND '1 benzothiophen 2 yl':ti,ab,kw AND 'methyl] 4 fluorophenyl] dextro glucitol':ti,ab,kw OR 'asp 1941':ti,ab,kw OR 'asp1941':ti,ab,kw OR suglat:ti,ab,kw

#18: #16 OR #17

#19: 'luseogliflozin'/exp OR 'luseogliflozin'

#20: '1, 5 anhydro 1 [5':ti,ab,kw AND '4 ethoxybenzyl':ti,ab,kw AND '2 methoxy 4 methylphenyl] 1 thio d glucitol':ti,ab,kw OR ('1, 5 anhydro 1 [5':ti,ab,kw AND '4 ethoxybenzyl':ti,ab,kw AND '2 methoxy 4 methylphenyl] 1 thio dextro glucitol':ti,ab,kw) OR ('1, 5 anhydro 1 [5':ti,ab,kw AND '4 ethoxybenzyl':ti,ab,kw AND '2 methoxy 4 methylphenyl] 1 thioglucitol':ti,ab,kw) OR ('2 [5':ti,ab,kw AND '4 ethoxybenzyl':ti,ab,kw AND '2 methoxy 4 methylphenyl] 6':ti,ab,kw AND hydroxymethyl:ti,ab,kw AND 'tetrahydrothiopyran 3, 4, 5 triol':ti,ab,kw) OR ('2 [5 [':ti,ab,kw AND '4 ethoxyphenyl':ti,ab,kw AND 'methyl] 2 methoxy 4 methylphenyl] 6':ti,ab,kw AND hydroxymethyl:ti,ab,kw AND 'thiane 3, 4, 5 triol':ti,ab,kw) OR lusefi:ti,ab,kw OR 'ts 071':ti,ab,kw OR 'ts071':ti,ab,kw

#21: #19 OR #20

#22: 'tofogliflozin'/exp OR 'tofogliflozin'

#23: 6:ti,ab,kw AND '4 ethylbenzyl':ti,ab,kw AND '3, 4, 5, 6 tetrahydro 6 hydroxymethylspiro [isobenzofuran 1':ti,ab,kw AND 3h:ti,ab,kw AND ', 2 pyran] 3, 4, 5 triol':ti,ab,kw OR ('6 [':ti,ab,kw AND '4 ethylphenyl':ti,ab,kw AND 'methyl] 3, 4, 5, 6 tetrahydro 6':ti,ab,kw AND hydroxymethyl:ti,ab,kw AND 'spiro [isobenzofuran 1':ti,ab,kw AND 3h:ti,ab,kw AND ', 2 [2h] pyran] 3, 4, 5 triol':ti,ab,kw) OR ('6 [':ti,ab,kw AND '4 ethylphenyl':ti,ab,kw AND 'methyl] 6':ti,ab,kw AND hydroxymethyl:ti,ab,kw AND '3, 4, 5, 6 tetrahydro 3h spiro [2 benzofuran 1, 2 pyran] 3, 4, 5 triol':ti,ab,kw) OR 'csg 452':ti,ab,kw OR 'csg452':ti,ab,kw OR 'rg 7201':ti,ab,kw OR 'rg7201':ti,ab,kw OR 'apleway':ti,ab,kw

#24: #22 OR #23

#25: 'sotagliflozin'/exp OR 'sotagliflozin'

#26: '2 [4 chloro 3':ti,ab,kw AND '4 ethoxybenzyl':ti,ab,kw AND 'phenyl] 6':ti,ab,kw AND methylthio:ti,ab,kw AND 'tetrahydro 2h pyran 3, 4, 5 triol':ti,ab,kw OR 'lp 802034':ti,ab,kw OR 'lp802034':ti,ab,kw OR 'lx 4211':ti,ab,kw OR 'lx4211':ti,ab,kw OR ('methyl 5 [4 chloro 3':ti,ab,kw AND '4 ethoxybenzyl':ti,ab,kw AND 'phenyl] 1 thio beta levo xylopyranoside':ti,ab,kw) OR ('methyl 5 [4 chloro 3 [':ti,ab,kw AND '4 ethoxyphenyl':ti,ab,kw AND 'methyl] phenyl] 1 thio beta levo xylopyranoside':ti,ab,kw) OR 'sar 439954':ti,ab,kw OR 'sar439954':ti,ab,kw OR zynquista:ti,ab,kw

#27: #25 OR #26

#28: 'remogliflozin etabonate'/exp OR 'remogliflozin etabonate'

#29: 4:ti,ab,kw AND '4 isopropoxybenzyl':ti,ab,kw AND '1 isopropyl 5 methyl 1h pyrazol 3 yl 6 o':ti,ab,kw AND ethoxycarbonyl:ti,ab,kw AND 'beta dextro glucopyranoside':ti,ab,kw OR ('5 methyl 4 [4':ti,ab,kw AND '1 methylethoxy':ti,ab,kw AND 'benzyl] 1':ti,ab,kw AND '1 methylethyl':ti,ab,kw AND '1h pyrazol 3 yl 6 o':ti,ab,kw AND ethoxycarbonyl:ti,ab,kw AND 'beta dextro glucopyranoside':ti,ab,kw) OR 'gsk 189075':ti,ab,kw OR 'gsk 189075a':ti,ab,kw OR 'gsk189075':ti,ab,kw OR 'gsk189075a':ti,ab,kw

#30: #28 OR #29

#31: 'sergliflozin etabonate'/exp OR 'sergliflozin etabonate'

#32: 2:ti,ab,kw AND '4 methoxybenzyl':ti,ab,kw AND 'phenyl 6 o':ti,ab,kw AND ethoxycarbonyl:ti,ab,kw AND 'beta dextro glucopyranoside':ti,ab,kw OR ('2 [':ti,ab,kw AND '4 methoxyphenyl':ti,ab,kw AND 'methyl] phenyl 6 o':ti,ab,kw AND ethoxycarbonyl:ti,ab,kw AND 'beta dextro glucopyranoside':ti,ab,kw) OR 'gw 869682':ti,ab,kw OR 'gw 869682x':ti,ab,kw OR

'gw869682':ti,ab,kw OR 'gw869682x':ti,ab,kw OR 'kgt 1251':ti,ab,kw OR 'kgt1251':ti,ab,kw OR sergliflozin:ti,ab,kw

#33: #31 OR #32

#34: #3 OR #6 OR #9 OR #12 OR #15 OR #18 OR #21 OR #24 OR #27 OR #30 OR #33

#35: 'metformin'/exp OR 'metformin'

#36: diaformin:ti,ab,kw OR aron:ti,ab,kw OR deson:ti,ab,kw OR dextin:ti,ab,kw OR diabetmin:ti,ab,kw OR diabetosan:ti,ab,kw OR diabex:ti,ab,kw OR '1, 1 dimethylbiguanide':ti,ab,kw OR flumamine:ti,ab,kw OR dimethylbiguanide:ti,ab,kw OR diformin:ti,ab,kw OR dimefor:ti,ab,kw OR dianben:ti,ab,kw OR dimethyldiguanide:ti,ab,kw OR dmgg:ti,ab,kw OR diamin:ti,ab,kw OR fortamet:ti,ab,kw OR glifage:ti,ab,kw OR glucomet:ti,ab,kw OR gluconil:ti,ab,kw OR glucophage:ti,ab,kw OR gluformin:ti,ab,kw OR glumet:ti,ab,kw OR glumetza:ti,ab,kw OR glupa:ti,ab,kw OR glyciphage:ti,ab,kw OR glycomet:ti,ab,kw OR glycon:ti,ab,kw OR glyformin:ti,ab,kw OR glymet:ti,ab,kw OR 'i max':ti,ab,kw OR 'la 6023':ti,ab,kw OR meguan:ti,ab,kw OR melbin:ti,ab,kw OR melformin:ti,ab,kw OR mellittin:ti,ab,kw OR metaformin:ti,ab,kw OR metformax:ti,ab,kw OR 'metformin hydrochloride':ti,ab,kw OR metformina:ti,ab,kw OR metformine:ti,ab,kw OR methformin:ti,ab,kw OR metiguanide:ti,ab,kw OR metphormin:ti,ab,kw OR 'n dimethylguanylguanidine':ti,ab,kw OR 'n, n dimethylbiguanidine':ti,ab,kw OR neoform:ti,ab,kw OR nndg:ti,ab,kw OR riomet:ti,ab,kw OR siofor:ti,ab,kw

#37: #35 OR #36

#38: 'diabetes mellitus'/exp OR 'diabetes mellitus'

#39: 'diabetic complication':ti,ab,kw OR 'diabetic obesity':ti,ab,kw OR 'experimental diabetes mellitus':ti,ab,kw OR 'impaired glucose tolerance':ti,ab,kw OR 'insulin dependent diabetes mellitus':ti,ab,kw OR 'lipoatrophic diabetes mellitus':ti,ab,kw OR ('maternally inherited diabetes':ti,ab,kw AND deafness:ti,ab,kw) OR 'newborn diabetes mellitus':ti,ab,kw OR 'non insulin dependent diabetes mellitus':ti,ab,kw OR 'pregnancy diabetes mellitus':ti,ab,kw OR 'wolfram syndrome':ti,ab,kw OR diabetes:ti,ab,kw OR diabetic:ti,ab,kw

#40: #38 OR #39

#41: #34 AND #37 AND #40

Cochrane Search Strategy:

#1：MeSH descriptor: [Sodium-Glucose Transporter 2] explode all trees

#2：("sodium glucose transporter 2"):ti,ab,kw OR ("slc5a2 protein"):ti,ab,kw OR ("sglt2 protein"):ti,ab,kw OR ("SGLT2I"):ti,ab,kw

#3：#1 or #2

#4: (dapagliflozin):ti,ab,kw OR (Farxiga):ti,ab,kw OR (Forxiga):ti,ab,kw OR ("BMS 512148"):ti,ab,kw OR ("BMS512148"):ti,ab,kw OR ("BMS-512148"):ti,ab,kw

#5: (Empagliflozin):ti,ab,kw OR ("BI 10773"):ti,ab,kw OR ("BI10773"):ti,ab,kw OR ("BI-10773"):ti,ab,kw OR (Jardiance):ti,ab,kw

#6: MeSH descriptor: [Canagliflozin] explode all trees

#7: (Invokana):ti,ab,kw OR ("Canagliflozin Hemihydrate"):ti,ab,kw

#8: #6 OR #7

#9: (ertugliflozin):ti,ab,kw OR (Steglatro):ti,ab,kw OR ("PF 04971729"):ti,ab,kw OR (PF04971729):ti,ab,kw OR ("PF-04971729"):ti,ab,kw

#10: (ipragliflozin):ti,ab,kw OR (Suglat):ti,ab,kw OR ("ASP1941"):ti,ab,kw OR ("ASP-1941"):ti,ab,kw

#11: (luseogliflozin):ti,ab,kw OR (Lusefi):ti,ab,kw OR ("TS 071"):ti,ab,kw OR ("TS-071"):ti,ab,kw

#12: (CSG452):ti,ab,kw OR (Apleway):ti,ab,kw OR (Deberza):ti,ab,kw OR (tofogliflozin):ti,ab,kw

#13: (sotagliflozin):ti,ab,kw OR ("LX4211"):ti,ab,kw OR ("LX-4211"):ti,ab,kw OR (zynquista):ti,ab,kw

#14: (remogliflozin etabonate):ti,ab,kw

#15: (sergliflozin etabonate):ti,ab,kw

#16: #3 OR #4 OR #5 OR #8 OR #9 OR #10 OR #11 OR #12 OR #13 OR #14 OR #15

#17: MeSH descriptor: [Metformin] explode all trees

#18: (Dimethylbiguanidine):ti,ab,kw OR ("dimethylguanylguanidine"):ti,ab,kw OR (Glucophage):ti,ab,kw OR ("Metformin Hydrochloride"):ti,ab,kw OR ("hydrochloride metformin"):ti,ab,kw OR ("Metformin HCl"):ti,ab,kw

#19: #17 OR #18

#20: MeSH descriptor: [Diabetes Mellitus] explode all trees

#21: ("diabetes mellitus experimental"):ti,ab,kw OR ("diabetes mellitus type 1"):ti,ab,kw OR ("Wolfram Syndrome"):ti,ab,kw OR ("diabetes mellitus type 2"):ti,ab,kw OR ("diabetes mellitus lipoatrophic"):ti,ab,kw OR ("diabetes gestational"):ti,ab,kw OR ("Diabetic Ketoacidosis"):ti,ab,kw OR ("Donohue Syndrome"):ti,ab,kw OR ("Latent Autoimmune Diabetes in Adults"):ti,ab,kw OR ("Prediabetic State"):ti,ab,kw OR ("Diabetes Complications"):ti,ab,kw OR ("Diabetic Angiopathies"):ti,ab,kw OR ("Diabetic Cardiomyopathies"):ti,ab,kw OR ("Diabetic Coma"):ti,ab,kw OR ("Diabetic Nephropathies"):ti,ab,kw OR ("Diabetic Neuropathies"):ti,ab,kw OR ("Fetal Macrosomia"):ti,ab,kw OR ("diabetes"):ti,ab,kw OR ("DM"):ti,ab,kw

#22: #20 OR #21

#23: #16 AND #19 AND #22

Web of science Search Strategy:

#1: TOPIC: (Sodium-Glucose Transporter 2) OR TOPIC: (sodium glucose transporter 2) OR TOPIC: (slc5a2 protein) OR TOPIC: (sglt2 protein) OR TOPIC: (SGLT2i)

#2: TOPIC: (dapagliflozin) OR TOPIC: (Farxiga) OR TOPIC: (Forxiga) OR TOPIC: ("BMS 512148") OR TOPIC: ("BMS512148") OR TOPIC: ("BMS-512148")

#3: TOPIC: (Empagliflozin) OR TOPIC: ("BI 10773") OR TOPIC: ("BI10773") OR TOPIC: ("BI-10773") OR TOPIC: (Jardiance)

#4: TOPIC: (Canagliflozin) OR TOPIC: (Invokana) OR TOPIC: ("Canagliflozin Hemihydrate")

#5: TOPIC: (ertugliflozin) OR TOPIC: (Steglatro) OR TOPIC: ("PF 04971729") OR TOPIC: ("PF04971729") OR TOPIC: ("PF-04971729")

#6: TOPIC: (ipragliflozin) OR TOPIC: (Suglat) OR TOPIC: ("ASP1941") OR TOPIC: ("ASP-1941")

#7: TOPIC: (luseogliflozin) OR TOPIC: (Lusefi) OR TOPIC: ("TS 071") OR TOPIC: ("TS-071")

#8: TOPIC: ("CSG452") OR TOPIC: (Apleway) OR TOPIC: (Deberza) OR TOPIC: (tofogliflozin)

#9: TOPIC: (sotagliflozin) OR TOPIC: ("LX4211") OR TOPIC: ("LX-4211") OR TOPIC: (zynquista)

#10: TOPIC: ("remogliflozin etabonate")

#11: TOPIC: ("sergliflozin etabonate")

#12: ((((((((((#1) OR #2) OR #3) OR #4) OR #5) OR #6) OR #7) OR #8) OR #9) OR #10) OR #11

#13: TOPIC: (Metformin) OR TOPIC: (Dimethylbiguanidine) OR TOPIC: (Dimethylguanylguanidine) OR TOPIC: (Glucophage) OR TOPIC: ("Metformin Hydrochloride") OR TOPIC: ("hydrochloride metformin") OR TOPIC: ("Metformin HCl")

#14: TOPIC: ("Diabetes Mellitus") OR TOPIC: ("diabetes mellitus experimental")

OR TOPIC: ("diabetes mellitus type 1") OR TOPIC: ("Wolfram Syndrome") OR

TOPIC: ("diabetes mellitus type 2") OR TOPIC: ("diabetes mellitus lipoatrophic")

OR TOPIC: ("diabetes gestational") OR TOPIC: ("Diabetic Ketoacidosis") OR TOPIC:

("Donohue Syndrome") OR TOPIC: ("Latent Autoimmune Diabetes in Adults") OR

TOPIC: ("Prediabetic State") OR TOPIC: ("Diabetes Complications") OR TOPIC: ("Diabetic Angiopathies") OR TOPIC: ("Diabetic Cardiomyopathies") OR TOPIC: ("Diabetic Coma") OR TOPIC: ("Diabetic Nephropathies") OR TOPIC: ("Diabetic Neuropathies") OR TOPIC: ("Fetal Macrosomia") OR TOPIC: (Diabetes) OR TOPIC:

("DM")

#15: #14 AND #13 AND #12

ClinicalTrials.gov Search Strategy:

Sodium-Glucose Transporter 2 | Studies With Results | Interventional Studies | Diabetes Mellitus

Also searched for Diabetes and Sodium Glucose Cotransporter. See Search Details

CNKI Search Strategy:

"Sodium-Glucose Transporter 2" and "Diabetes Mellitus" and "Metformin" translated into Chinese

Wanfang Search Strategy:

"Sodium-Glucose Transporter 2" and "Diabetes Mellitus" and "Metformin" translated into Chinese
